# Microbial community structure and functional potential in a long-term uranium–nickel contaminated ecosystem

**DOI:** 10.3389/fmicb.2026.1741152

**Published:** 2026-01-28

**Authors:** Christian Chukwujindu, Max Kolton, Olasunkanmi Fasakin, Ashish Pathak, John Seaman, Ashvini Chauhan

**Affiliations:** 1 School of the Environment, Florida A&M University, Tallahassee, FL, United States; 2 French Associates Institute for Agriculture and Biotechnology of Drylands, Ben-Gurion University of the Negev, Beersheba, Israel; 3 Savannah River Ecology Laboratory, University of Georgia, Aiken, SC, United States

**Keywords:** antibiotic resistance genes, heavy metal contamination, metal resistance genes, mobile genetic elements, nickel, uranium

## Abstract

This study examined the microbial community structure, functional potential, and resistance determinants in uranium (U)- and nickel (Ni)-contaminated soils from the Savannah River Site (SRS), a former nuclear materials production and waste collection facility operated by the U. S. Department of Energy (DOE). Soil cores were collected from the Steed Pond area, where long-term discharge of acidic wastewater resulted in spatially variable contamination levels. Concentrations of U and Ni in the collected samples ranged from 0.22–10.44 g kg^−1^ and 0.79–2.28 g kg^−1^, respectively. Shotgun metagenomic and high-throughput quantitative PCR (HT-qPCR) analyses revealed bacterial communities dominated by Pseudomonadota, Actinomycetota, and Acidobacteriota, with enrichment of taxa affiliated with genera known to include diazotrophic members (e.g., *Bradyrhizobium* and *Burkholderia*), alongside increased abundance of nitrogen fixation–related functional genes. Carbon and nitrogen cycle genes were generally well represented across samples, with selective shifts observed in acetate assimilation genes (acsA/acsE) and comparatively low abundance of hydrazine oxidoreductase (hzo), indicating pathway-specific variation rather than broad metabolic suppression. A total of 117 resistance-associated genes were identified, comprising 93 antibiotic-resistance genes (ARGs), 3 metal-resistance genes (MRGs), and 21 mobile genetic elements (MGEs). Strong positive correlations among ARGs, MRGs, and MGEs indicate co-selection and horizontal gene transfer, forming a genetically mobile resistome. Collectively, these findings demonstrate that long-term U–Ni contamination selects for metabolically versatile, diazotroph-enriched, and genetically mobile microbiomes. Such communities exhibit both resistance proliferation and bioremediation potential, providing key insights into microbial adaptation and ecosystem recovery in legacy nuclear-contaminated soils.

## 1 Introduction

Microorganisms play a crucial role in maintaining soil health, regulating nutrient cycle, decomposing organic matter, and ensuring soil structural stability (Wang et al., 2024). They also transform and immobilize toxic substances, thereby contributing to natural attenuation and *in situ* soil bioremediation (Simonoff et al., 2007). Consequently, a diverse and functionally robust microbial community is essential for maintaining soil quality and serves as a sensitive bioindicator of ecosystem health (Rogiers et al., 2021).

Anthropogenic activities, particularly mining and industrial operations, can profoundly disrupt soil microbial diversity and function through the deposition of heavy metals and radionuclides (International Atomic Energy Agency, 2004; United Nations Scientific Committee on the Effects of Atomic Radiation, 1993). Heavy metal contamination alters microbial growth, morphology, and metabolism (Song et al., 2018), leading to shifts in community composition that make microbial communities effective early indicators of environmental pollution (Li et al., 2017; Theodorakopoulos et al., 2017).

A key consequence of heavy metal contamination is the proliferation of multidrug-resistant (MDR) microorganisms, which compromise the efficacy of antibiotics and pose a growing public-health risk (San Millan, 2018; World Health Organization, 2023). This phenomenon is primarily explained by co-selection, in which genes conferring metal and antibiotic resistance are physically or functionally linked, often on mobile genetic elements (MGEs), which facilitates their joint horizontal transfer (Zhang et al., 2025). Under metal stress, this linkage accelerates the persistence and spread of antibiotic-resistance genes (ARGs) and metal-resistance genes (MRGs) in soil ecosystems, forming a highly mobile resistome under combined chemical pressures (Pal et al., 2015).

Microorganisms mitigate heavy metal toxicity using diverse physiological and biochemical mechanisms. These include the use of efflux pumps that expel toxic ions, enzymatic detoxification (e.g., phosphatase-mediated precipitation of uranium as insoluble U-phosphate complexes), and secretion of extracellular polymeric substances (EPS) that chelate or immobilize metals (Sprocati and Alisi, 2018). These strategies enhance microbial survival under metal stress and affect contaminant mobility and bioavailability, thereby affecting natural attenuation processes in soils.

The Savannah River Site (SRS) in South Carolina is a historically contaminated U. S. Department of Energy (DOE) facility where uranium (U) and nickel (Ni) are the dominant heavy metal pollutants (Pathak et al., 2017). From 1960 to 1985, acidic wastewater from the M-Area reactor complex was discharged into the Tim’s Branch–Steed Pond system and eventually flowed downstream into Upper Three Runs Creek (Evans et al., 1992; Edwards et al., 2014). During site operation, approximately 44 tons of depleted and natural uranium (U) and comparable quantities of nickel (Ni) were released (Murray et al., 2010). Although containment basins were constructed to limit contaminant migration, the low-gradient topography of Tim’s Branch–Steed Pond, coupled with acidic wastewater effluents, promoted sedimentation and enhanced mobilization of U and Ni. As a result, soil and groundwater U and Ni contamination reached concentrations exceeding 1 g/kg at Steed Pond (Millings and Amidon, 2013; Evans et al., 1992; Punshon et al., 2003). Such contamination can inhibit the activity of enzymes involved in the carbon, nitrogen, phosphorus, and sulfur (CNPS) cycle, thereby disrupting organic matter decomposition, nutrient availability, and greenhouse gas fluxes.

Previous rRNA gene amplicon-based studies at SRS and other DOE sites (Oak Ridge Reservation (ORR) and the Old Rifle Processing Site) revealed strong associations between heavy metal concentrations and antibiotic resistance gene abundance (Thomas et al., 2020; Pathak et al., 2020). However, functional predictions in these studies relied on computational inference (e.g., PICRUSt), which requires experimental validation (Thomas et al., 2020; Pathak et al., 2020). While metagenomics provides valuable insights into taxonomic and potential functional shifts, it often yields only relative, rather than quantitative, data on gene abundance (Abramova and Bengtsson-Palme, 2025).

High-throughput quantitative PCR (HT-qPCR) overcomes these limitations by enabling the simultaneous quantification of hundreds of functional genes, including ARGs, MGEs, MRGs, and biogeochemical cycle markers, such as those involved in nitrogen fixation (Zhang and Dick, 2014). Nitrogen-fixing (diazotrophic) microorganisms are of particular interest in metal-contaminated soils because they can supply bioavailable nitrogen under nutrient-limited conditions and often harbor metal-resistance determinants (Hu et al., 2024). Genera such as *Bradyrhizobium*, *Burkholderia*, and *Rhodanobacter* are frequently reported from U- and Ni-contaminated sites, suggesting that diazotroph enrichment may represent an adaptive response to chronic heavy metal stress (Verma and Kuila, 2019; Pathak et al., 2020; Jaswal et al., 2019a, 2019b). HT-qPCR has also been successfully applied to groundwater and soils impacted by anthropogenic contamination, providing robust quantitative data on ARGs, MGEs, and MRGs (Silvester et al., 2025).

Despite extensive research at Steed Pond, the integrated taxonomic and functional characterization of its microbiome remains incomplete. Therefore, this study aimed to: (i) characterize the microbial community composition of U- and Ni-contaminated soils; (ii) quantify the abundance and evaluate response of functional genes involved in carbon, nitrogen, phosphorus, and sulfur (CNPS) cycle to metal gradients; and (iii) examine the co-occurrence of ARGs, MRGs, and MGEs as indicators of co-selection processes. We hypothesize that long-term U–Ni contamination (i) suppresses carbon- and nitrogen-cycle functional genes due to chronic metabolic stress and energetic trade-offs, and (ii) enriches antibiotic resistance genes via co-selection with metal resistance genes and enhanced horizontal gene transfer mediated by mobile genetic elements.

## 2 Materials and methods

### 2.1 Site description

#### 2.1.1 The Savannah River site and the steed pond area

The Savannah River Site (SRS), a former nuclear weapons production facility located in South Carolina, spans approximately 800 km^2^ and has been managed by the U. S. Department of Energy since the 1950s (U.S. Environmental Protection Agency, 2021). The site produced tritium and plutonium-237. Between 1960 and 1985, acidic wastewater from a fuel- and aluminum-clad uranium reactor was discharged into the Tim’s Branch–Steed Pond system, eventually reaching Upper Three Runs Creek (Edwards et al., 2014; Supplementary Figure S1). During this period, the Steed Pond area received up to 44,000 kg of depleted and natural uranium, along with comparable amounts of nickel (Murray et al., 2010), creating long-term heavy-metal and radionuclide gradients that are ideal for studying microbial adaptation and co-selection mechanisms.

Ten samples were selected to represent a spatially resolved uranium–nickel contamination gradient within the Steed Pond area. Sampling locations were selected based on historical discharge pathways, proximity to the Tim’s Branch inflow, and prior geochemical surveys that indicated heterogeneous metal distributions across the site. The selected samples span a wide range of uranium (0.22–10.44 g kg^−1^) and nickel (0.79–2.28 g kg^−1^) concentrations, enabling comparative analysis across low, intermediate, and highly contaminated conditions.

All samples consisted of surface soils collected from the upper soil horizon (0–15 cm) and were treated consistently as soils rather than sediments. Sites were spatially distinct from one another, capturing both geochemical and microbial heterogeneity across the contamination gradient. This gradient-based sampling design enabled the assessment of how increasing U–Ni burden influences microbial community structure, functional gene abundance, and the co-selection of resistance determinants.

### 2.2 Sample collection

Ten soil and sediment samples were collected from the Steed Pond area (Supplementary Figure S1) using a manual JMC 18″ soil core sampler to a depth of 15 cm. Surface plant detritus was removed prior to sampling. Each core was placed in sterile polyethylene tubes, kept in an ice chest during transport, and subsequently stored at −20 °C until processing. All procedures were conducted in accordance with standard laboratory radiation safety protocols. Samples were labeled S0001B through S0010B.

### 2.3 Analysis of heavy metals and physicochemical properties

Approximately 0.5 g of homogenized soil was digested in 10 mL of concentrated HNO₃ following EPA Method 6020B (U.S. Environmental Protection Agency, 2014; Hu et al., 2017). Uranium (U) and nickel (Ni) total concentrations were quantified in duplicate using an inductively coupled plasma mass spectrometer, with a detection limit of 0.001–100 ppm (ICP-MS; Perkin Elmer, Optima 8000IC) (U.S. EPA, 2007). Analytical accuracy and precision were verified by analyzing instrument blanks and certified reference materials after every 10 samples. The physicochemical parameters analyzed included organic carbon, pH, moisture, total carbon (TC), total phosphorus (TP), and total nitrogen (TN). Loss on ignition was carried out to determine the total organic matter content of the samples using the method described by Robertson (2011). The total carbon and nitrogen contents were determined using the AOAC Official Method 972.43 (University of California, Davis, Analytical Laboratory, 2025). The total phosphorus and the pH were determined using the modified EPA method (Li et al., 2021).

### 2.4 Shotgun-based metagenomics

Total genomic DNA was extracted from Steed Pond soil cores using the DNeasy PowerLyzer Kit (QIAGEN) with 0.5 g of the core samples, following the manufacturer’s protocol. DNA concentration and purity were evaluated using a NanoDrop 2000 micro-volume spectrophotometer (Thermo Fisher Scientific, Wilmington, DE, United States) prior to the preparation of the metagenomic library. Purified DNA libraries were sequenced on an Illumina HiSeq 2000 platform (2 × 100 bp paired end). Raw reads were quality-filtered and trimmed using Trimmomatic v0.36 to remove adapters, ambiguous bases, and reads shorter than 100 bp. Only reads with >80% bases at Q ≥ 30 were retained for downstream analysis (Agashe et al., 2024). Sequence quality metrics are provided in Supplementary Table S1. High-quality reads were assembled using MEGAHIT and classified taxonomically through the Pathosystems Resource Integration Center^1^ and the One-Codex database platform^2^ pipelines (Agashe et al., 2024). Taxonomic assignments in PATRIC were performed with the Kraken2 classifier, which uses taxon-specific k-mers for alignment-free read classification. Extraction blanks were not included in the DNA extraction process for this study. To mitigate potential contamination, downstream analyses focused on genes and taxa with consistent detection across samples, and low-abundance features were excluded that were unlikely to represent true soil-associated signals.

### 2.5 Metagenome annotation

Assembled metagenomic sequences were annotated using the Metagenome Rapid Annotation Subsystem Technology pipeline.^3^ Gene prediction and functional assignment were performed against MG-RAST’s integrated databases, including the SEED Subsystems (Overbeek et al., 2005) and KEGG orthology (Kanehisa et al., 2016) modules. Protein-coding genes were identified using default parameters, and relative gene-abundance profiles were generated for each sample. Functional distributions were visualized using pie-donut charts to illustrate the proportional contribution of metabolic categories across datasets.

### 2.6 HT-qPCR quantification of microbial genes

High-throughput quantitative PCR (HT-qPCR) was used to quantify the abundance of functional genes associated with carbon, nitrogen, phosphorus, and sulfur (CNPS) cycle, as well as antibiotic-, metal-, and mobile-resistance determinants (ARGs, MRGs, and MGEs). A total of 456 validated primer sets (Zheng and Ding, 2019; Yan et al., 2024) targeted the 16S rRNA gene, 35 carbon-, 22 nitrogen-, 9 phosphorus-, 5 sulfur-cycle genes, 309 antibiotic resistance genes (ARGs) covering all major antibiotic classes, 57 mobile genetic elements (MGEs) including insertion sequences, integrases, transposases, and plasmid-associated genes, 10 metal resistance genes (MRGs), and 8 taxonomic marker genes (Supplementary Table S2).

Each 100 μL reaction mixture contained 1 × SYBR Green I Master Mix, 1–3 ng of DNA template, and 1 μM of each primer. Reactions were run on a SmartChip Real-Time PCR System (Takara). The thermal cycle consisted of initial enzyme activation (95 °C, 10 min), followed by 40 cycles of denaturation (95 °C, 30 s), annealing (58 °C for CNPS genes or 60 °C for 384 ARG/MGE/MRG genes, 30 s), and an extension (72 °C, 30 s). Melting curve analysis was automatically generated, and the qPCR data were analyzed using the SmartChip qPCR software.

Reactions were performed in triplicate with non-template controls. Wells with amplification efficiencies outside 1.7–2.3 or R^2^ < 0.99 were excluded, and those with a threshold cycle (CT) value below 31 were considered positive. Relative gene copy numbers were calculated using the method described by Looft et al. (2012) and expressed in Equation 1.


$$\text{Relative Copy Number}=10^{\left(31-\mathrm{CT})/(10÷3\right)}$$ (1)

where CT refers to the Threshold Cycle, and 31 refers to the detection limit.

High-throughput quantitative PCR (HT-qPCR) data were initially generated as absolute gene copy numbers based on standard curves and are reported per gram of dry soil. Prior to downstream analyses, gene abundances were normalized to 16S rRNA gene copy numbers to account for variation in microbial biomass across samples (Supplementary Table S3). Log₁₀ transformation was applied to all gene copy data to stabilize variance and accommodate the wide dynamic range (8–10 orders of magnitude) inherent to HT-qPCR datasets. The HT-qPCR panel targets a curated subset of known metal resistance genes and is not intended to provide exhaustive coverage of all metal resistance mechanisms present in environmental metagenomes.

### 2.7 Metagenomic sequence accession number

Shotgun metagenomic sequences have been deposited in the NCBI Sequence Read Archive (SRA) under BioProject accession number PRJNA816857.

## 3 Results

### 3.1 Heavy metal concentrations and soil physicochemical properties of the steed pond area

**Figure 1:**
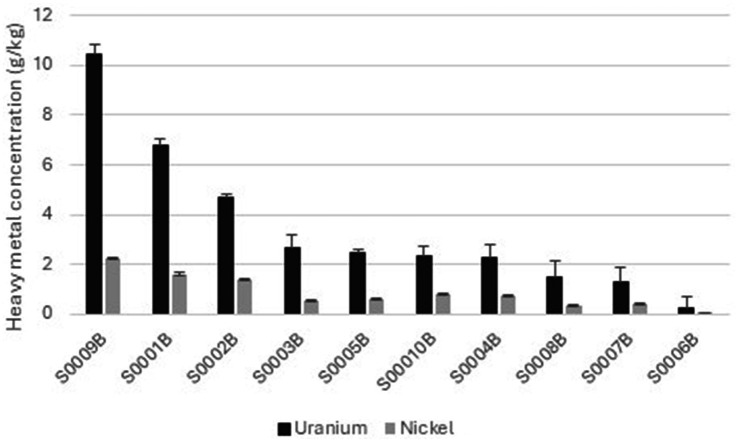
Concentrations of uranium (U) and nickel (Ni) across soil cores collected from the Steed Pond area of SRS. Heavy-metal concentrations were quantified in duplicate using inductively coupled plasma mass spectrometry (ICP-MS). Error bars represent the standard deviation between replicate measurements. Samples with elevated U concentrations also exhibited higher Ni levels, suggesting co-contamination from a common industrial source and similar geochemical mobility within the sediment profile.

Uranium (U) concentration ranged from 0.223 to 10.439 g/kg, while the nickel (Ni) concentration ranged from 0.079 to 2.2 g/kg across the ten soil cores (Figure 1). Samples S0009B exhibited the highest U and Ni levels (30 and 26%, respectively), whereas S0006B had the lowest levels (6 and 9%, respectively; Figure 1). A significant positive correlation exists between U and Ni concentrations (*r* = 0.98, *p* < 0.05; Supplementary Figure S2), demonstrating their co-occurrence and suggesting a common industrial source and similar geochemical mobility within the Steed Pond system. The results of the soil physicochemical parameters are shown in Supplementary Table S4. It is notable that the site is rich in phosphorus, with values ranging from 540 to 1,137 mg/kg. The site is slightly and uniformly acidic with pH values ranging from 4.5 to 4.8. The average moisture content, organic matter, soil pH, total carbon, total nitrogen, and total phosphorus were 61.4 ± 4.0%, 35.5 ± 13.0%, 4.6 ± 0.13, 214.6 ± 67.8 mg/kg, 9.2 ± 4.0 mg/kg, and 949 ± 170 mg/kg, respectively.

### 3.2 Taxonomic classification of the microbial community in the steed pond area

**Figure 2:**
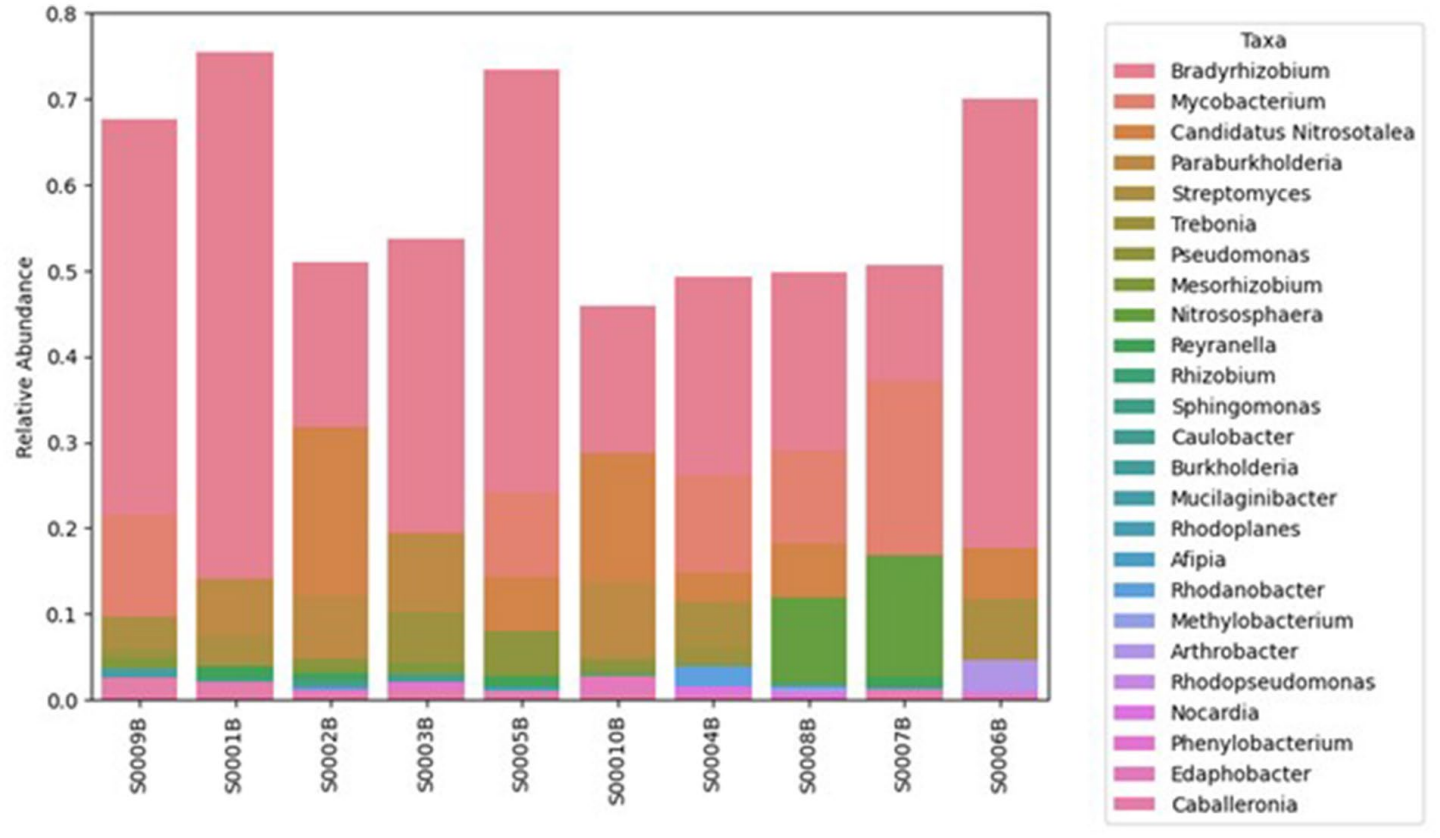
Relative abundance of identified microbial genera in soil samples from the Steed Pond area of SRS. Metagenomic-based taxonomic classification was conducted, and only reads confidently assigned at the genus level are shown in this graph. The samples are presented in order of decreasing uranium concentration. The corresponding phylum-level composition is presented in Supplementary Figure S3, and the complete genus-level profile in Supplementary Figure S4.

Shotgun metagenomics was used to characterize the microbial taxonomic composition in the Steed Pond area. Summary statistics for total sequence reads and assembly metrics are provided in Supplementary Table S1. Pseudomonadota (78 ± 2%), Actinomycetota (16 ± 3%), and Acidobacteriota (4 ± 1%) were the dominant phyla, accounting for approximately 98% of all classified bacterial reads. The bacterial abundance at the phylum level is shown in Supplementary Figure S3. Community structure analysis highlighted the high abundance of putative nitrogen-fixing (diazotrophic) populations. Among these, *Bradyrhizobium* (6.2 ± 2%) was the most abundant across all samples, followed by *Paraburkholderia* (2.9 ± 1%), *Burkholderia* (2.0 ± 1%), *Rhodopseudomonas* (2.2 ± 1%; phototrophic diazotroph), *Caulobacter* (1.6 ± 1%), *Mesorhizobium* (1.5 ± 1%; symbiotic diazotroph), *Devosia* (1.5 ± 0.5%), and *Afipia* (1.4 ± 1%). Collectively, these putative diazotrophs accounted for 20 ± 3% of the total prokaryotic community (Supplementary Figure S4; Figure 2). Figure 2 includes only genera with confident taxonomic assignment; unclassified or low-abundance genera (“no genus,” “others”) are shown in Supplementary Figure S4. The dominance of these nitrogen-fixing taxa, together with the presence of *Mycobacterium* (5.1 ± 2%), a genus commonly identified in heavy metal-contaminated soils (Verma and Kuila, 2019), suggests that long-term exposure to U and Ni may drive localized nitrogen limitation, selecting for metal-tolerant diazotrophic populations. The alpha and beta diversity indices are presented in Supplementary Figure S5. The alpha diversity indices, measured by the Shannon diversity index, range from 5.9 to 7.3. The PCA plot of the beta diversity indices, based on the Bray–Curtis dissimilarity matrix, indicates that the S0009B sample, with the highest contamination level, is distinct from the other samples.

### 3.3 Metagenome annotation and functional analysis

Functional annotation based on the SEED subsystem database revealed the dominance of genes involved in carbohydrate metabolism (15%), clustering-based subsystems (15%), amino acid metabolism (13%), protein metabolism (13%), and membrane transport (7%) (Supplementary Figure S6A). These categories accounted for more than 60% of all annotated functions. The enrichment of carbohydrate metabolism and oxidative stress–response genes likely reflects microbial strategies to sustain high energy demand and facilitate detoxification and cellular repair under high uranium and nickel exposure (Yan et al., 2016).

**Figure 3:**
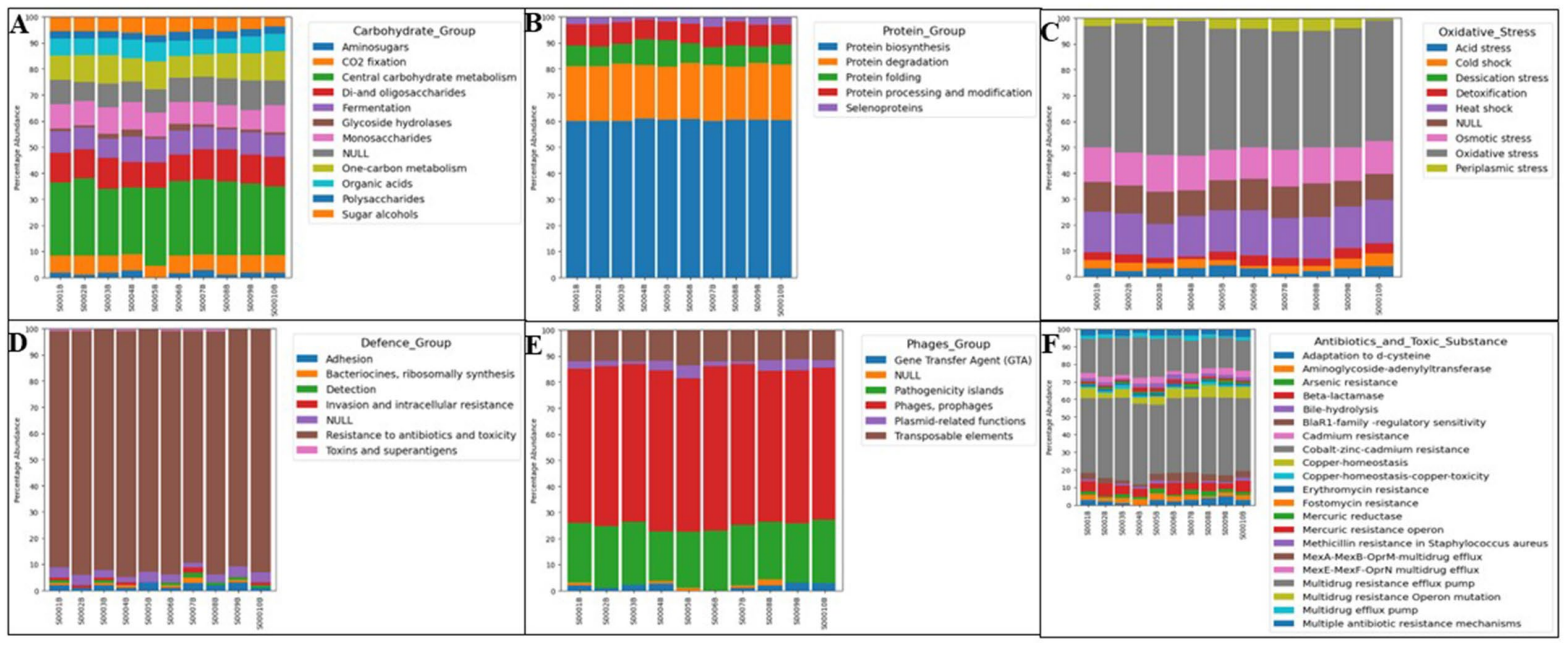
Functional characterization of metagenomic sequences from the Steed Pond area of SRS using MG-RAST subsystem classification. Each bar represents an individual soil sample (S0001 B–S00010 B), and colors indicate the relative contribution of specific functional categories within each sample. At subsystem level II, the major functions were associated with central carbohydrate metabolism **(A)**, protein biosynthesis **(B)**, oxidative stress **(C)**, resistance to antibiotics and toxic substances **(D)**, phages, prophages, and transposable elements **(E)**, and multidrug and metal resistance **(F)**. The subsystem level 1 classification is shown in Supplementary Figure S6.

At SEED Subsystem Level II, functions associated with membrane transport, antibiotic and metal resistance, and mobile genetic elements (MGEs) were enriched (Figure 3). Functions related to phages and transposases suggest active horizontal gene transfer, consistent with the co-selection of resistance determinants reported for other metal-impacted soils (Partridge et al., 2018).

**Figure 4:**
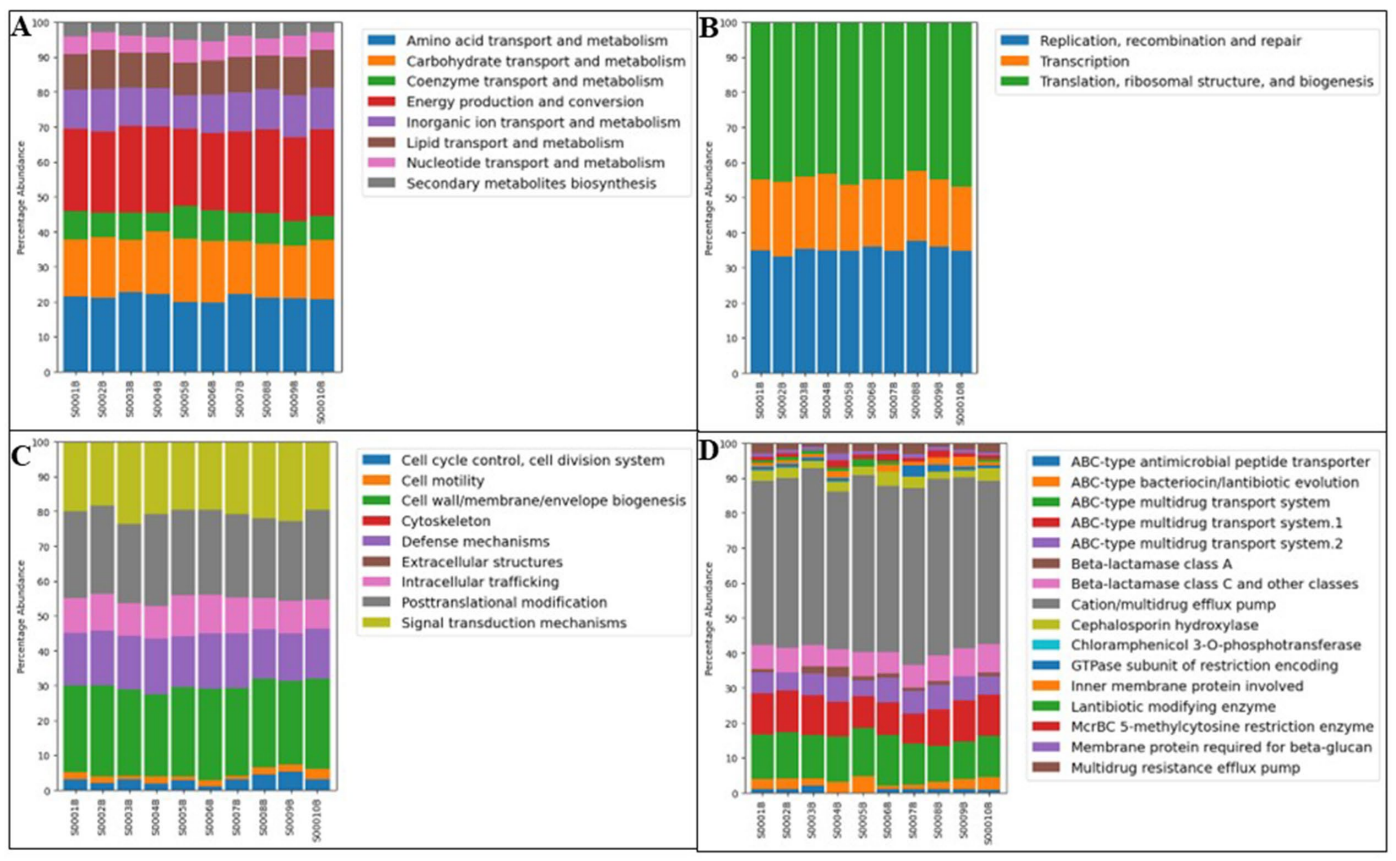
Functional characterization of metagenomic sequences from the Steed Pond area of SRS based on Clusters of Orthologous Groups (COG) classification. Metagenomic functional profiles were annotated using the MG-RAST platform and organized according to COG functional categories. Showing are the genes related to metabolism **(A)**, information storage and processing **(B)**, cellular processing and signaling **(C)**, and defense mechanisms **(D)**. The COD main categories are shown in Supplementary Figure S6.

Similarly, the functional classification of Clusters of Orthologous Groups (COG) also indicates that metabolism-related genes account for ~50% of all annotated functions, followed by cellular processes and signaling genes (19%), and information storage and processing genes (17%) (Supplementary Figure S6B; Figure 4). Moreover, genes related to defense mechanisms (~20%), including multidrug-efflux transporters and genes conferring resistance to beta-lactams, aminoglycosides, and chloramphenicol, were also enriched (Figure 4). Collectively, these observations highlight the intense selective pressures shaping the community and support the hypothesis that energy-intensive metabolic versatility and genetic mobility support microbial survival under stressful conditions.

### 3.4 Distribution of CNPS cycle genes

A total of 72 functional genes involved in the carbon (C), nitrogen (N), phosphorus (P), and sulfur (S) cycles were detected across the soil cores. Nearly all functional genes involved in CNPS cycle were detected, except for the hydrazine oxidase gene (*hzo*) (Supplementary Figure S7). Absolute abundances of CNPS genes ranged from approximately 10^8^ to 10^1^⁰ copies/g soil: Specifically, carbon-cycle genes ranged from 4 × 10^9^ to 2 × 10^10^ copies/g, nitrogen-cycle from 9 × 10^8^ to 5 × 10^9^, phosphorus-cycle from 7 × 10^8^ to 4 × 10^9^, and sulfur-cycle from 7 × 10^8^ to 4 × 10^9^ copies/g soil (Supplementary Figure S8).

The abundance of carbon cycle genes was generally consistent across samples, except that *acsA* and *acsE* genes, which play crucial roles in acetate assimilation (Zheng et al., 2025), were more abundant in less contaminated samples. This suggests U and Ni contamination suppress carbon cycle activity, specifically pathways associated with acetate metabolism or anaerobic processes that function under low redox potential. The high absolute abundance of carbon cycle genes (10^9^–10^1^⁰ copies g^−1^ dry soil) is consistent with the dominance of bacterial populations involved in core metabolic processes and reflects the cumulative contribution of multiple gene copies across diverse taxa in soils with high microbial biomass and organic matter content, rather than overestimation of individual functional capacity. Such values fall within the upper range reported for bulk soil metagenomic and qPCR studies when genes involved in central metabolism are quantified on a per–dry–soil–mass basis (Tian et al., 2025).

Nitrogen cycle genes were well represented, including *nifH* (nitrogen fixation), *ureC* (ammonification), *gdhA* (mineralization), *amoB* (nitrification), and *nosZ1*/*nosZ2* (denitrification). Their abundances ranged from 10^6^ to 10^9^ copies/g soil, with *amoB*, *gdhA*, *nosZ2*, and *ureC* among the most abundant (8 × 10^8^ ± 2–5 × 10^8^ copies/g soil). In contrast, the hydrazine oxidoreductase genes (*hzo*), which serve as biomarkers for detecting ammonium-oxidizing bacteria, were relatively low (Supplementary Figure S8B). This pattern indicates that nitrogen fixation, rather than ammonium oxidation, is the principal nitrogen acquisition strategy under long-term U–Ni stress.

### 3.5 Distribution of antibiotic–and metal-resistance genes, and mobile genetic elements

A total of 117 resistance-associated genes were detected across all soil cores, including 93 antibiotic resistance genes (ARGs), 3 metal resistance genes (MRGs), and 20 mobile genetic elements (MGEs), in addition to the 16S rRNA gene used for normalization. ARG abundances varied widely, ranging from approximately 8 × 10^5^ to 8 × 10^1^⁰ copies/g of soil across multiple antibiotic resistance gene (ARG) classes (Supplementary Figures S9–S10).

Multidrug resistance genes were the most prevalent category, with 31 genes detected, and *qepA*_1_2 being the most abundant (6.0 × 10^8^ ± 3 copies/g soil). Among aminoglycoside resistance genes (23 total), *aac3*-via was the most abundant (4.0 × 10^8^ ± 5.0 copies/g soil), while *blaOXY*-1 (3.0 × 10^8^ ± 3 copies/g soil) dominated the beta-lactam class (11 genes). Additional resistance determinants were detected for fluoroquinolones, macrolide-lincosamide-streptogramin (MLSB), phenicols, and tetracyclines (10 genes in total), with *cmIV* showing the highest abundance (2.0 × 10^6^ ± 3.0 copies/g soil). Genes conferring resistance to glycopeptides, peptides, sulfonamides, diaminopyrimidines, and rifamycins (GPSDR; 17 genes) were also detected, with *vanA* (4.0 × 10^5^ ± 3 copies/g soil) and *fabK* (5.0 ± 0.00 × 10^5^ copies/g soil) being the most abundant representatives for glycopeptides and other subgroups, respectively (Supplementary Figures S8–S9).

A total of 20 MGEs were detected, with *trb-C* being the most prevalent (3 × 10^8^ ± 3 copies/g soil; Supplementary Figure S11). These elements facilitate horizontal gene transfer, contributing to the spread of resistance traits within microbial communities. The MGEs were mostly insertion sequences (54%) and plasmid replication proteins (14%), based on the total gene copy numbers per gram of soil. The classification of the MGEs is summarized in Supplementary Table S5. Three MRGs conferring resistance to arsenic (*arsC*), lead (*pbrA*), and copper (*copA*) were also detected. Among them was *copA*, encoding a copper-translocating P-type ATPase that helps maintain copper homeostasis in bacterial cells (Rensing and Mitra, 2007), which was most abundant (3.0 × 10^8^ ± 3.0 copies/g soil) (Supplementary Figure S11). Collectively, the co-occurrence of diverse ARGs, MGEs, and MRGs indicates a highly mobile resistome structured by long-term U–Ni exposure, where selective pressure from heavy metals may have promoted horizontal transfer and persistence of antibiotic resistance determinants.

### 3.6 Correlation among metal resistance genes, antibiotic resistance genes, mobile genetic elements, and soil physicochemical properties

**Figure 5:**
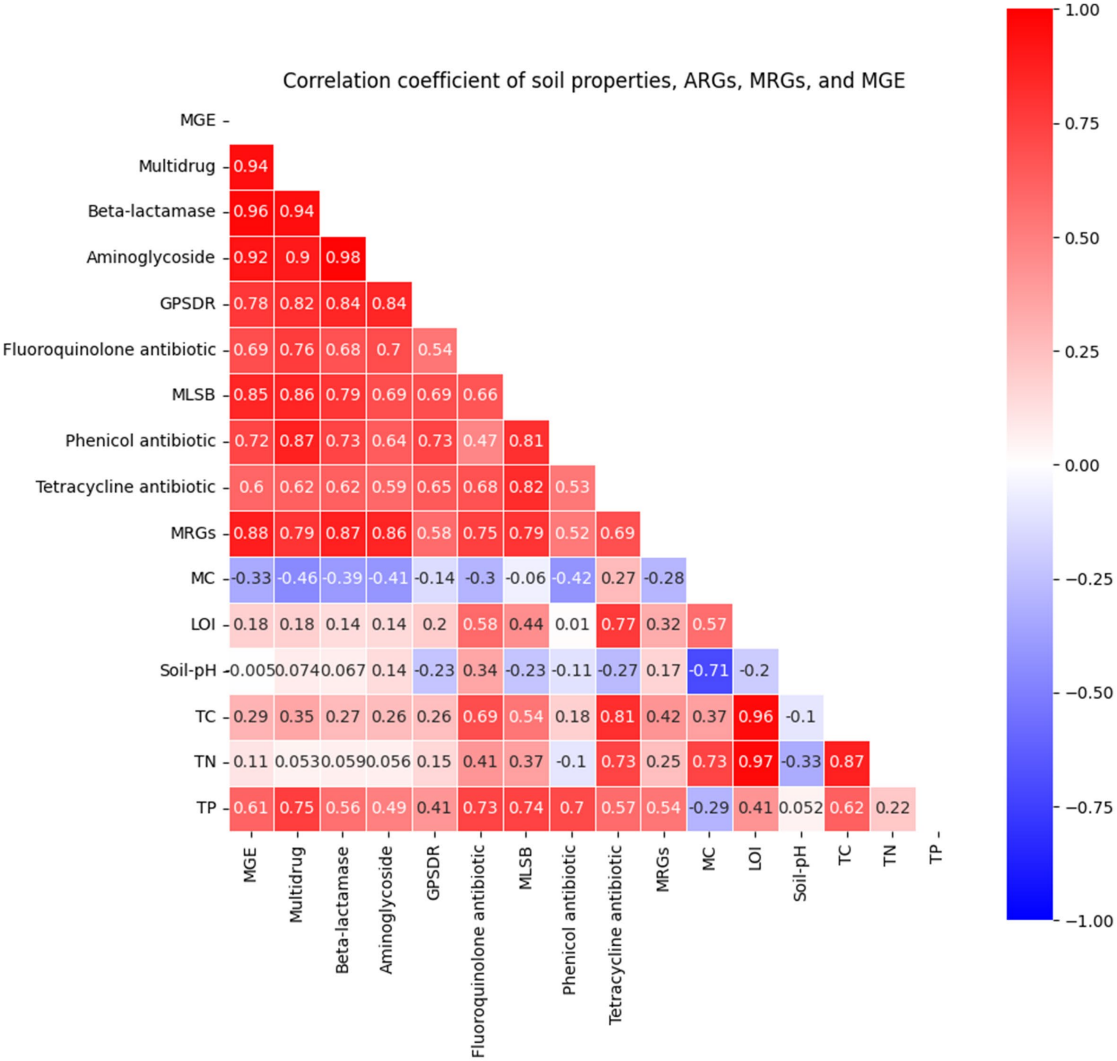
Correlation matrix among antibiotic-resistance genes (ARGs), metal-resistance genes (MRGs), and mobile genetic elements (MGEs), and physicochemical properties in soils from the Steed Pond area of SRS. Correlations were calculated using Pearson’s correlation coefficient (*r*) based on gene abundances obtained from high-throughput quantitative PCR (HT-qPCR). Warmer colors (red) indicate positive correlations, while cooler colors (blue) indicate negative correlations. Strong positive associations were observed between MGEs and several ARG classes, particularly *β*-lactamase (*r* = 0.96), multidrug-resistance genes (0.94), and aminoglycoside (*r* = 0.92) resistance genes, as well as between MRGs and ARGs, suggesting that long-term U–Ni contamination promotes the co-selection and horizontal dissemination of resistance determinants. These relationships demonstrate the formation of a genetically interconnected and mobile resistome shaped by chronic exposure to heavy metals. On the other hand, the soil’s physicochemical properties showed a weak correlation with the ARGs, MRGs, and MGEs, indicating that the proliferation of these resistance genes was not driven by the soil’s physicochemical properties.

The relative abundance of mobile genetic elements (MGEs) was positively correlated with both metal-resistance genes (MRGs) (*r* = 0.88) and some classes of antibiotic-resistance genes (ARGs) (Figure 5). Notably, MGEs exhibited strong correlations with aminoglycoside (*r* = 0.92) and *β*-lactamase (*r* = 0.96) resistance genes, suggesting that these ARGs are preferentially mobilized under heavy-metal stress (Figure 5). Positive associations were also observed between MRGs and specific ARG classes, particularly β-lactamase (*r* = 0.87) and aminoglycoside (*r* = 0.86) resistance genes (Figure 5). Furthermore, inter-ARG correlations were detected, with the β-lactamase–aminoglycoside pair showing a strong positive correlation (*r* = 0.98; Figure 5). These overlapping relationships suggest that ARGs, MRGs, and MGEs are genetically and functionally linked, forming an integrated resistome shaped by chronic U–Ni exposure. Such patterns are consistent with previous metagenomic studies showing that β-lactamase, macrolide-lincosamide-streptogramin (MLSB), and aminoglycoside resistance genes are among the most recurrent ARG classes in metal-impacted soils, often co-localized with MGEs and MRGs on plasmids or transposons (Forsberg et al., 2014; Gou et al., 2018; Hu et al., 2017). On the other hand, the physicochemical properties did not show a strong correlation with the resistance genes (Figure 5). Some of the physicochemical properties showed strong interconnections; notably, total organic matter was strongly correlated with total carbon (TC) (*r* = 0.96) and total nitrogen (TN) (*r* = 0.97), while the soil pH exhibited a strong negative correlation with moisture content (*r* = −0.71). However, the physicochemical parameters were generally not associated with resistance genes; approximately 85% of the pairwise absolute correlation coefficients were less than 0.6.

The Mantel test, which applies permutation to establish the correlation between two distance matrices, was carried out in RStudio to confirm the association among the MGE, MRGs, ARG, and the soil physicochemical properties. The results of the Mantel test, with 999 permutations, indicate that the Pearson correlation coefficient between the ARGs and MGEs was 0.913(*p*-value = 0001); ARGs and MRGs = 0.698 (*p*-value = 0.001); MGEs and MRGs = 0.74 (*p*-value = 0.001); and ARGs and Soil Physicochemical properties = −0.039 (*p*-value = 0.502). The summary of the Mantel test is shown in Supplementary Table S6.

Collectively, these findings demonstrate that long-term U–Ni contamination drives the formation of genetically mobile resistomes, where metal-induced selective pressure enhances both the co-occurrence and horizontal dissemination of antibiotic- and metal-resistance genes across microbial populations.

## 4 Discussion

This study demonstrates that long-term uranium (U) and nickel (Ni) contamination at the Savannah River Site (SRS) is associated with the enrichment of metal-tolerant microbial taxa and a diverse, mobile resistome in Steed Pond soils. Taxonomic profiles were dominated by Pseudomonadota, Actinomycetota, and Acidobacteriota, phyla commonly reported in metal-contaminated environments and known for broad metabolic versatility and stress tolerance. These findings are consistent with prior observations from metalliferous soils and support the view that chronic metal exposure selects for microbial groups capable of persisting under sustained geochemical stress (Jaswal et al., 2019a; Thomas et al., 2020).

Functional gene analyses indicate that the potential for carbon and nitrogen cycles is largely maintained across the contamination gradient, with pathway-specific variation rather than broad suppression. Carbon cycle genes were generally comparable among samples, although acetate assimilation genes (*acsA* and *acsE*) were more abundant in less contaminated soils, suggesting that specific anaerobic or low-redox carbon pathways were subject to selective constraints under elevated U–Ni conditions (Zheng et al., 2025). Nitrogen cycle genes, encompassing fixation, mineralization, nitrification, and denitrification, were widely distributed and abundant, while hydrazine oxidoreductase (*hzo*) genes were consistently low, indicating differential sensitivity among nitrogen transformation pathways.

Taxa affiliated with genera that include known diazotrophic members were enriched in contaminated soils, accompanied by elevated abundances of nitrogen fixation–related genes. Because nitrogen fixation is strain-specific, these results are interpreted as reflecting increased diazotrophic potential rather than genus-wide functionality. Under chronic metal stress, these taxa may gain a competitive advantage by maintaining their nitrogen acquisition capacity despite the elevated energetic demands associated with detoxification and stress response mechanisms.

A prominent feature of the Steed Pond microbiome was the extensive co-occurrence of antibiotic resistance genes (ARGs) and mobile genetic elements (MGEs). We detected 93 ARGs and 20 MGEs spanning multiple antibiotic classes, with strong correlations indicative of horizontal gene transfer and long-term maintenance of resistance traits. In contrast, only three canonical metal resistance genes (*arsA, copA*, and *pbrT*) were detected. This limited detection likely reflects the targeted scope of the HT-qPCR panel rather than an absence of metal resistance capacity, as uranium and nickel resistance are frequently mediated by indirect mechanisms, such as sequestration, efflux systems with broad specificity, redox transformation, and extracellular immobilization, which are not captured by gene-targeted assays (Rogiers et al., 2021).

Collectively, these results indicate that long-term U–Ni contamination promotes microbial communities characterized by metabolic flexibility, selective retention of key nutrient cycle functions, and a genetically mobile resistome. Rather than wholesale functional collapse, chronic metal stress appears to act as an ecological filter favoring taxa and genetic traits that balance energetic costs with persistence. This adaptive configuration likely contributes to microbial resilience in legacy nuclear-contaminated soils, with implications for natural attenuation and bioremediation potential at long-term contaminated sites.

## 5 Conclusion

Heavy metal contamination at the Savannah River Site (SRS) altered the distribution of microbial functional genes, reducing ecological functionality, while enriching resistance determinants. The strong positive correlations among antibiotic-resistance genes (ARGs), metal-resistance genes (MRGs), and mobile genetic elements (MGEs) highlight the risk that such contamination may amplify the environmental spread of antibiotic resistance, posing a significant public health concern. Nevertheless, the enrichment of resistance taxa and functional genes also suggests that these microbial assemblages could be leveraged for bioremediation of contaminated environments.

## Data Availability

The datasets presented in this study can be found in online repositories. The names of the repository/repositories and accession number(s) can be found at: https://www.ncbi.nlm.nih.gov/genbank/, PRJNA816857.

## Footnotes

1 PATRIC, https://www.patricbrc.org

2
https://www.onecodex.com/

3 MG-RAST; www.mgrast.org
