# Supplementary material for: Microbial community structure and functional potential in a long-term uranium–nickel contaminated ecosystem
Source: Front Microbiol. 2026 Jan 28;17:1741152. doi: 10.3389/fmicb.2026.1741152 (PMC12893718; doi:10.3389/fmicb.2026.1741152)
Supplement: Supplementary file 1 [file Supplementary_file_1.docx]

**Supplementary materials**


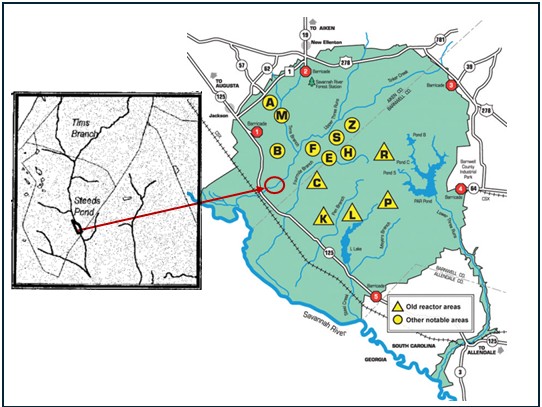


**Supplementary Figure S1.** Map of the Savannah River Site (SRS) showing major tributaries, reactor areas, and associated facilities. The figure illustrates the spatial layout of SRS, including key hydrological features and reactor locations relevant to historical uranium and nickel contamination. SRS depicting tributaries, reactors, and facilities. Source: <https://www.srs.gov/general/tour/online.htm>.


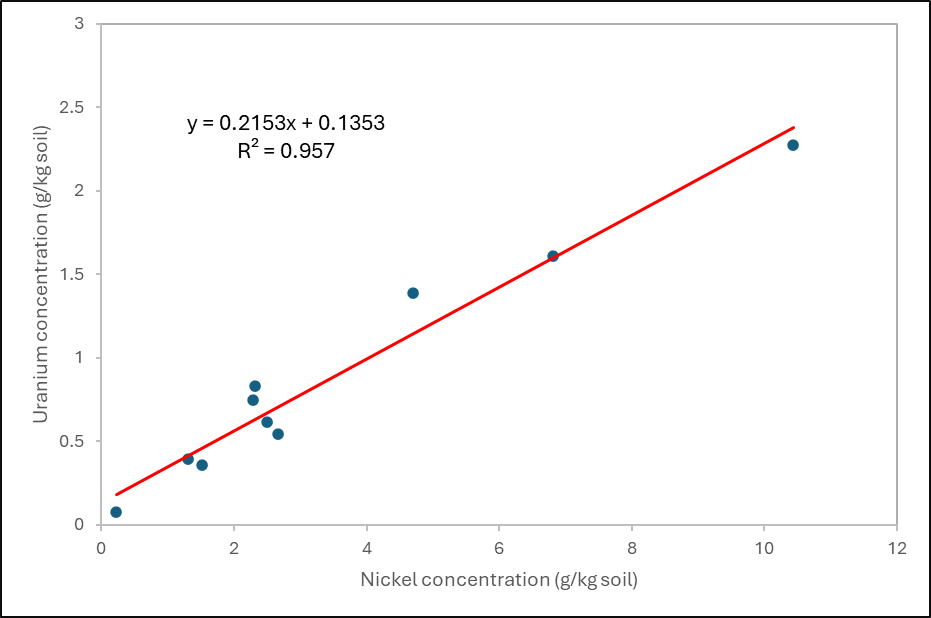


Pearson’s Correlation: 0.98

**Supplementary Figure S2.** Correlation between uranium (U) and nickel (Ni) concentrations in soil samples from the Steed Pond area of SRS. Uranium and nickel concentrations exhibited a strong positive linear relationship (Pearson’s r = 0.98), indicating co-contamination and shared geochemical behavior across sampling locations. The red line represents the best-fit linear regression, and each point corresponds to an individual soil sample.

**
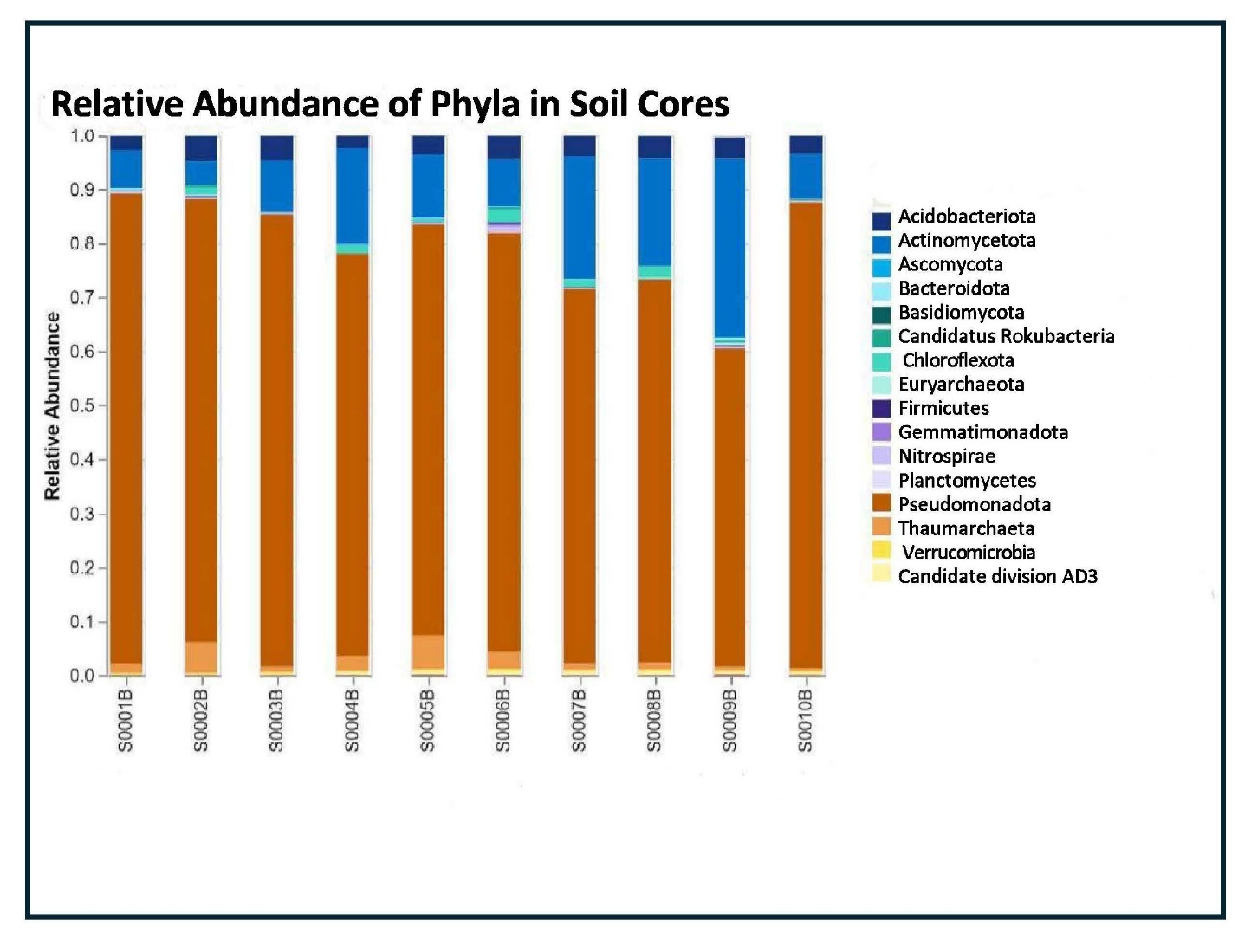
**

**Supplementary Figure S3.** Relative abundance of prokaryotic phyla in soil samples from the Steed Pond area of SRS. Taxonomic composition was determined using shotgun metagenomic sequencing. The plot shows the relative abundance of major bacterial phyla across individual soil cores (S0001B–S00010B). Each bar represents an individual sample, and the stacked colors indicate the proportional contribution of each phylum to the total bacterial community.


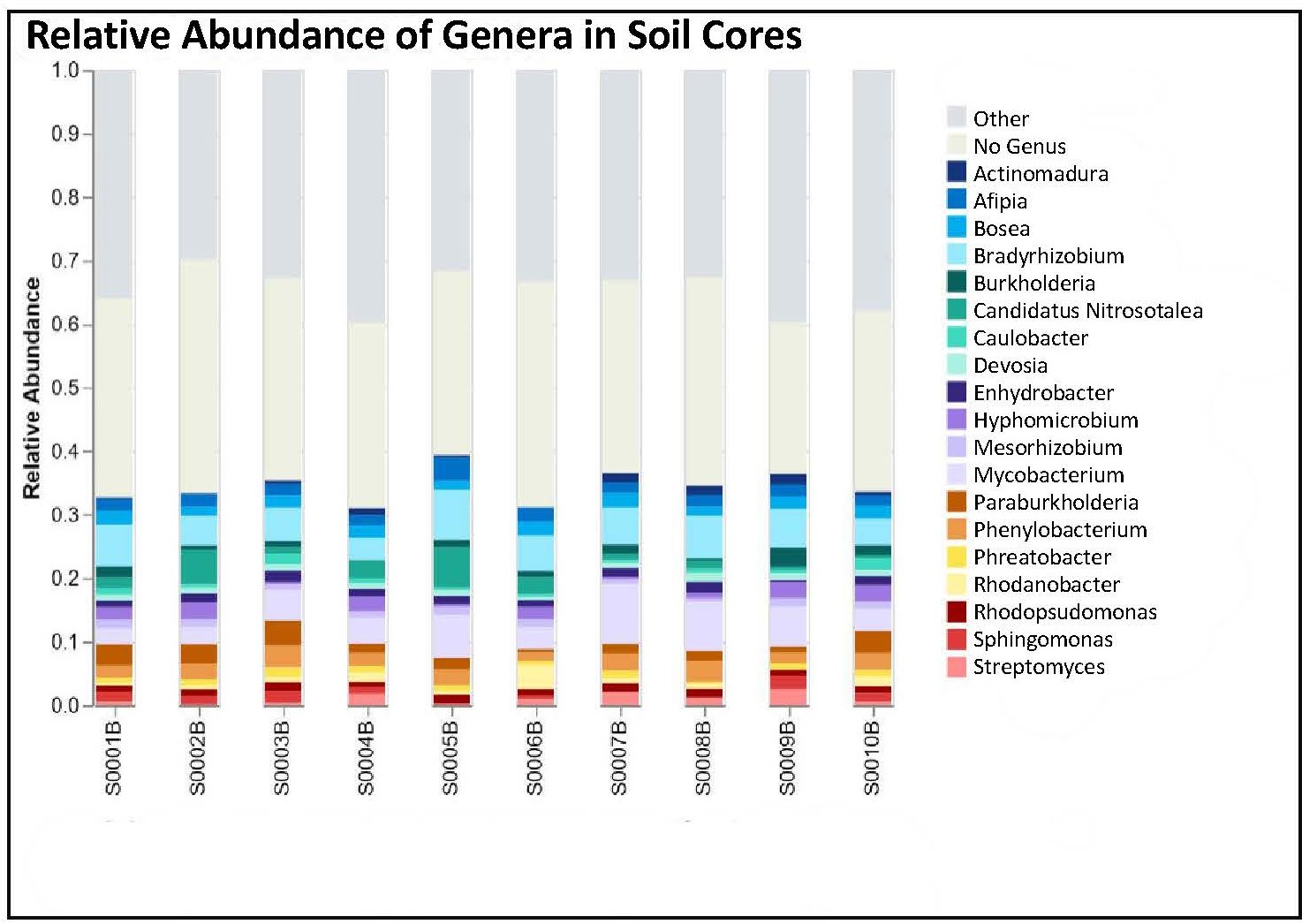


**Supplementary Figure S4.** Relative abundance of prokaryotic genera in soil samples from the Steed Pond area of SRS. Taxonomic composition was determined using shotgun metagenomic sequencing. The stacked-bar plot illustrates the relative abundance of dominant bacterial genera across soil cores (S0001B–S0010B). Each bar represents an individual soil sample, and colors indicate the proportional contribution of each genus to the total bacterial community. Unclassified or low-confidence taxa (“no genus,” “others”) accounted for more than 60% of total reads across samples.


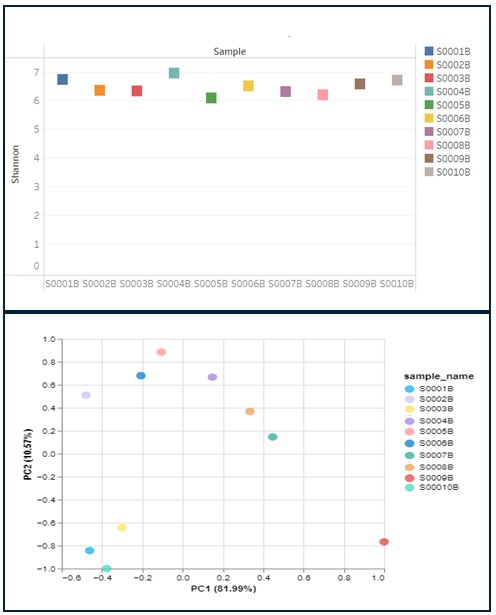


**Supplementary Figure S5.** The alpha diversity and beta diversity indices of the bacterial community at the genus level in the soil core samples collected from the Steed Pond area of SRS. The alpha diversity was measured by the Shannon diversity index, and the beta diversity was plotted on PCA using the Bray-Curtis dissimilarity matrix. The alpha diversity indices range from 5.9 to 7.3. The S0004B and S0001B had the highest alpha diversity indices. The beta diversity result shows the S0009B sample, which has the highest commination level, separated from the other samples.

**
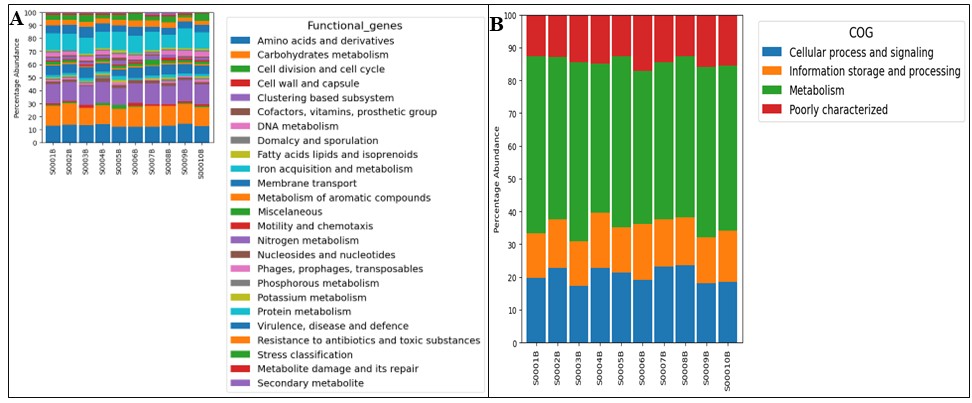
**

**Supplementary Figure S6**. The subsystem level 1 functional genes (A), and the categories of Clustered Orthologous Groups (COG)(B) at the Steed Pond area of SRS. The dominant subsystems are carbohydrate metabolism (15%), clustering-based subsystems (15%), amino acid metabolism (13%), protein metabolism (13%), and membrane transport (7%). With the COD system (B), the metabolism-related genes account for ~50% of all annotated functions, followed by cellular processes and signaling genes (19%), and information storage and processing genes (17%).


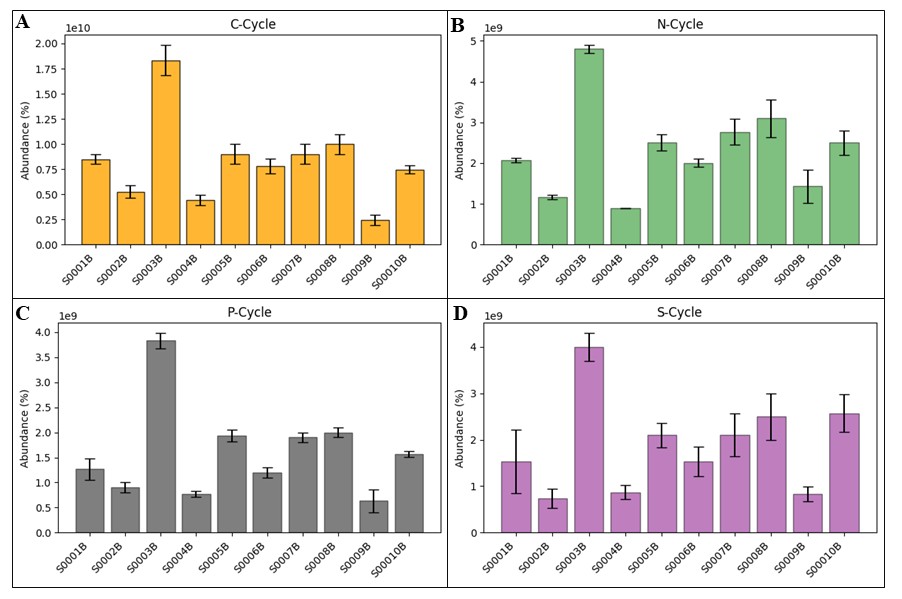


**Supplementary Figure S7.** Absolute copy numbers of CNPS (carbon, nitrogen, phosphorus, and sulfur) cycle genes in soil samples from the Steed Pond area of SRS.

Gene copy numbers were quantified using high-throughput quantitative PCR (HT-qPCR). Each bar represents the cumulative contribution of all genes belonging to each functional category analyzed within individual soil samples (C-cycle: $\left( 8.6\pm4.2 \right)\times{10}^{9}$genes/gsoil; N-cycle: $\left( 2.1\pm1.1 \right)\times{10}^{9}$genes/gsoil; P-cycle: $\left( 1.8\pm0.9 \right)\times{10}^{9}$genes/gsoil; S-cycle: $\left( 1.7\pm0.8 \right)\times{10}^{9}$genes/gsoil). The carbon-cycle genes were the most abundant across all samples.

**A**


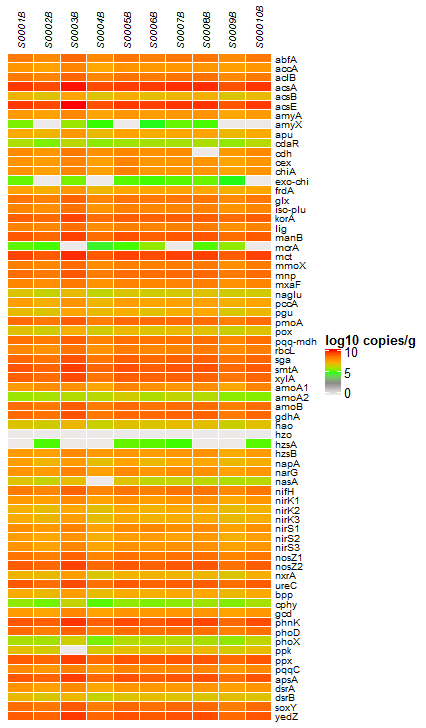

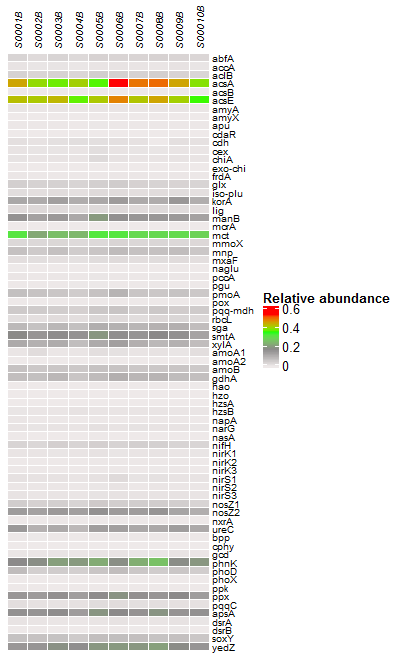


**B**

**Supplementary Figure S8.** Absolute (A) and relative (B) gene copy numbers of 72 CNPS (carbon, nitrogen, phosphorus, and sulfur) cycle genes in soil samples from the Steed Pond area of SRS. Values were determined using high-throughput quantitative PCR (HT-qPCR). Panel A shows the absolute abundance of each functional gene expressed as log₁₀ copies g⁻¹ soil, while Panel B presents the relative abundance normalized within each sample. The *acsA* and *acsE* genes, key components of the *acetyl-CoA* pathway for carbon assimilation, were more abundant in less-contaminated sites, suggesting a shift in carbon-metabolism potential along the uranium–nickel gradient.


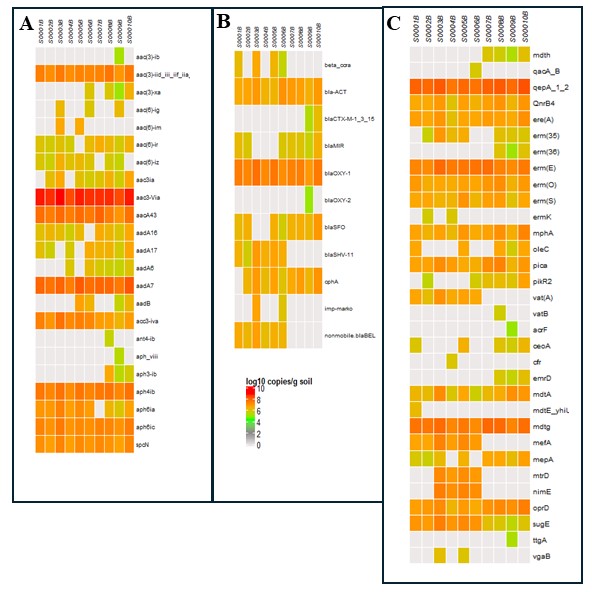


**Supplementary Figure S9.** Distribution of antibiotic resistance genes (ARGs) conferring resistance to aminoglycosides (A), β-lactams (B), fluoroquinolones, macrolide–lincosamide–streptogramin B (MLSB), and multidrug classes (C) in soil samples from the Steed Pond area of SRS. A total of 23 aminoglycosides, 11 β-lactamases, and 31 multidrug-resistance genes, along with fluoroquinolone and MLSB resistance genes, were detected by high-throughput quantitative PCR (HT-qPCR). The figure illustrates the relative abundance (log₁₀ gene copies g⁻¹ soil) of each ARG category across all samples. The dominance of β-lactamase and multidrug-resistance genes indicates strong selective pressure associated with heavy-metal contamination.


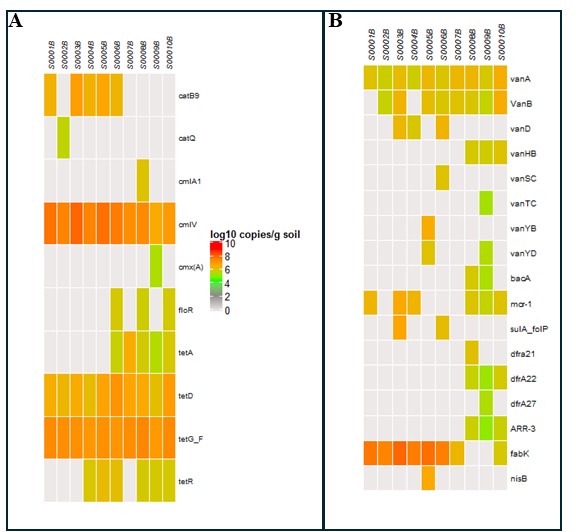


**Supplementary Figure S10.** Antibiotic resistance genes (ARGs) conferring resistance to phenicol and tetracycline (10 genes)(A) and to glycopeptide, peptide, sulfonamide, diaminopyrimidine, and rifamycin (GPSDR)(B) were detected in soil samples from the Steed Pond area of SRS. ARG copy numbers were quantified using high-throughput quantitative PCR (HT-qPCR) and are expressed as log₁₀ copies g⁻¹ soil. Each column represents an individual soil sample, and each row corresponds to a specific resistance gene. The *cmlIV*, *tetD*, and *fabK* genes were among the most abundant, indicating active selection for multidrug and cell-wall-associated resistance under long-term uranium–nickel contamination.


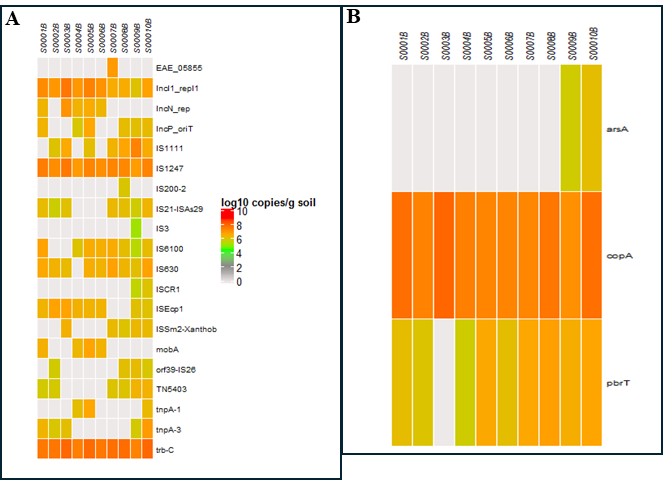


**Supplementary Figure S11.** Distribution of mobile genetic elements (MGEs) and metal-resistance genes (MRGs) in soil samples from the Steed Pond area of SRS. MGEs (A) and MRGs (B) were quantified using high-throughput quantitative PCR (HT-qPCR) and are expressed as log₁₀ gene copies g⁻¹ soil. The MGE panel includes insertion sequences (IS elements), transposases, integrases, plasmid replication and transfer genes, whereas the MRG panel includes *arsA*, *copA*, and *pbrT*, which confer resistance to arsenic, copper, and lead, respectively. Each column represents an individual soil sample, and each row corresponds to a specific gene. The widespread presence of MGEs and MRGs indicates strong potential for horizontal gene transfer and co-selection under uranium–nickel contamination gradients.

**Supplementary Tables**

**Supplementary** **Table S1.** Summary of assembled shotgun metagenomic sequence reads from soil samples collected at the Steed Pond area of SRS. Filtered and trimmed reads with a quality score > Q30 were assembled using MEGAHIT. The total number of assembled contigs per sample ranged from 774 to 8,681, reflecting variability in sequencing depth and assembly complexity among samples.

| Sample **ID** | **Raw Reads** | **%GC** | **Filtered Reads** | **%GC** | **Number of assembled Sequences** |
| --- | --- | --- | --- | --- | --- |
| S0001B | 2,181,138 | 59 | 1,948,120 | 58.8 | 840 |
| S0002B | 1,915,964 | 58.7 | 1,696,542 | 58.5 | 1374 |
| S0003B | 1,443,496 | 59.3 | 1,290,334 | 59.1 | 1215 |
| S0004B | 2,145,216 | 59.4 | 1,888,646 | 59.2 | 8681 |
| S0005B | 1,953,968 | 58.6 | 1,735,918 | 58.5 | 4791 |
| S0006B | 2,043,156 | 59.3 | 1,819,190 | 59.1 | 898 |
| S0007B | 1,655,466 | 59.7 | 1,459,730 | 59.5 | 2542 |
| S0008B | 1,541,652 | 59.9 | 1,367,960 | 59.7 | 5231 |
| S0009B | 2,325,258 | 59 | 2,057,192 | 58.8 | 774 |
| S00010B | 1,868,990 | 58.9 | 1,654,898 | 58.7 | 1685 |

**Supplementary Table S2.** The primer sets for elemental cycle and antibiotic resistance gene analysis

|  | **Gene Target** | **Type** | **Forward sequence (5′ → 3′)** | **Reverse sequence (5′ → 3′)** | **Ref** |
| --- | --- | --- | --- | --- | --- |
| 1 | *cdh* | Carbon Cycle (Cellulose Hydrolysis) | ATWRYCTWCCGMRTHGCCMT | GTKAGSGGRTTBYKGRYCAT | Zheng et al., 2019 |
| 2 | *cex* | Carbon Cycle(Cellulose Hydrolysis) | YSTACGGSATGCACTGGMT | TANCGCAGRTAGTCVCCCAT | Zheng et al., 2019 |
| 3 | *naglu* | Carbon Cycle (Cellulose Hydrolysis) | TVAAYTGGTAYCTGAAATAY | CCRTGYAGVGCCATCCAGTC | Zheng et al., 2019 |
| 4 | *chiA* | Carbon Cycle (Chitin Hydrolysis) | TSAAGAARTACGCSGACAACG | ASGTCATCAGRCCCTTSAG | Zheng et al., 2019 |
| 5 | *exo-chi* | Carbon Cycle (Chitin Hydrolysis) | GATTGGTSVCAATATGAYRG | STCCARCCACCRAYRCTRAA | Zheng et al., 2019 |
| 6 | *accA* | Carbon Cycle (Fixation) | GAAGGCTAYCGCAARGC | CCTTCMGGSGARATMAC | Zheng et al., 2019 |
| 7 | *aclB* | Carbon Cycle (Fixation) | TGGACMATGGTDGCYGGKGGT | ATAGTTKGGSCCACCTCTTC | Zheng et al., 2019 |
| 8 | *acsA* | Carbon Cycle (Fixation) | GATACCTGGTGGCAGACCGA | TGATCACGTCGTCGACCCGG | Zheng et al., 2019 |
| 9 | *acsB* | Carbon Cycle (Fixation) | CTYTGYCAGTCMTTYGCBCC | CCCATAAABCCYGGDGTYTG | Zheng et al., 2019 |
| 10 | *acsE* | Carbon Cycle (Fixation) | TCATCGGCGAACGCATCAAC | AGRCCGGCTTCSATGGC | Zheng et al., 2019 |
| 11 | *cdaR* | Carbon Cycle (Fixation) | CGARATGGTGGTGCTCAA | CARCGTRTTACGATGAATA | Zheng et al., 2019 |
| 12 | *frdA* | Carbon Cycle (Fixation) | MTGCTGCACACSCTGTW | CCGGTSGGGTGRWACTG | Zheng et al., 2019 |
| 13 | *korA* | Carbon Cycle (Fixation) | GCCGGCTACCCCATCACCCC | ATGATGGGATGGTCGCCATG | Zheng et al., 2019 |
| 14 | *mcrA* | Carbon Cycle (Fixation) | GGTGGTGTMGGDTTCACMCARTA | CGTTCATBGCGTAGTTVGGRTAGT | Zheng et al., 2019 |
| 15 | *mct* | Carbon Cycle (Fixation) | TGGGCGCSGASGTSATMCG | TTGACSGTRTARTCSAYSGC | Zheng et al., 2019 |
| 16 | *pccA* | Carbon Cycle (Fixation) | GTGMTGATCAAGGCCWC | CGSGTGTTCATYTCSAGGAA | Zheng et al., 2019 |
| 17 | *rbcL* | Carbon Cycle (Fixation) | AAGGAYGACGAGAACATC | TGCAGSATCATGTCRTT | Zheng et al., 2019 |
| 18 | *smtA* | Carbon Cycle (Fixation) | TTTCTGGCCGGBTAYGCDGC | CGGTACGGHCCGGTYTGVCC | Zheng et al., 2019 |
| 19 | *abfA* | Carbon Cycle (Hemicellulose Hydrolysis) | CGSTAYCCSGGCGGCAAYTT | TGCCASGGNCCGTCCATYTC | Zheng et al., 2019 |
| 20 | *manB* | Carbon Cycle (Hemicellulose Hydrolysis) | ATGCGCGGBGTCAACCA | TCGTTGSCGATGTTGABGA | Zheng et al., 2019 |
| 21 | *xylA* | Carbon Cycle (Hemicellulose Hydrolysis) | TGGGGBGGTCGYGAAGG | ACTTTGGCRTCRAAGTT | Zheng et al., 2019 |
| 22 | *glx* | Carbon Cycle (Lignin Hydrolysis) | AACCAGTCGATCATCTACGA | RTGSACGAGCTCDGGCATGG | Zheng et al., 2019 |
| 23 | *lig* | Carbon Cycle (Lignin Hydrolysis) | CCGCACACACTGTTGCTGC | CGAAGGATTGCCACTCGCA | Zheng et al., 2019 |
| 24 | *mnp* | Carbon Cycle (Lignin Hydrolysis) | MACRCCSTTCGACTCSACC | ACGTCSGAGCAGTCRAYGA | Zheng et al., 2019 |
| 25 | *pox* | Carbon Cycle (Lignin Hydrolysis) | ACYAGTATCCATTGGCACGGT | AGATGVGARTGATACCARAA | Zheng et al., 2019 |
| 26 | *mmoX* | Carbon Cycle (Methane Meabolism;oxidation) | ATGGAGGCGGTCAAGGACGA | CGCTTCATGCCCTTCCACAG | Zheng et al., 2019 |
| 27 | *pmoA* | Carbon Cycle (Methane Metabolism;oxidation) | GGNGACTGGGACTTCTGG | GAASGCNGAGAAGAASGC | Zheng et al., 2019 |
| 28 | *mxaF* | Carbon Cycle (Methane Metabolism;production) | GCGGCACCAACTGGGGCTGGT | GGGCAGCATGAAGGGCTCCC | Zheng et al., 2019 |
| 29 | *pqq-mdh* | Carbon Cycle (Methane Metabolism;production) | TGTTCTATGTGCCGGCCAA | CTTCCACAGTTCCTTGCC | Zheng et al., 2019 |
| 30 | *pgu* | Carbon Cycle (Pectin Hydrolysis) | ANCATTGGTGGCCSTGGAA | TTRAYGGCRATRCARTCRTC | Zheng et al., 2019 |
| 31 | *amyA* | Carbon Cycle (Starch Hydrolysis) | YGGTTTTCGTCTTGACGCSG | MGGCTGMGTRTCATGRTTK | Zheng et al., 2019 |
| 32 | *amyX* | Carbon Cycle (Starch Hydrolysis) | TATAAYTGGGGMTATGAYCC | CCCATYAAATCAAAWCGRAA | Zheng et al., 2019 |
| 33 | *apu* | Carbon Cycle (Starch Hydrolysis) | ACVTGGATAGGYGAGCCYCA | CCRTCSGGGAAGTAGTTKCC | Zheng et al., 2019 |
| 34 | *iso-plu* | Carbon Cycle (Starch Hydrolysis) | GTCATYTACTTYGGNCC | CGNGCSACATCNGCCCA | Zheng et al., 2019 |
| 35 | *sga* | Carbon Cycle (Starch Hydrolysis) | CGSAACTGGGAYTACCGS | TCCCACAGSCCSKCGTC | Zheng et al., 2019 |
| 36 | *ureC* | Nitrogen Cycle (Ammonification) | AAGMTSCACGAGGACTGGGG | AGRTGGTGGCASACCATSAGCAT | Zheng et al., 2019 |
| 37 | *hzo* | Nitrogen Cycle (Anaerobic Ammonium Oxidation) | AAGACNTGYCAYTGGGGWAAA | GACATACCCATACTKGTRTANACNGT | Zheng et al., 2019 |
| 38 | *hzsA* | Nitrogen Cycle (Anaerobic Ammonium Oxidation) | WTYGGKTATCARTATGTAG | AAABGGYGAATCATARTGGC | Zheng et al., 2019 |
| 39 | *hzsB* | Nitrogen Cycle (Anaerobic Ammonium Oxidation) | ARGGHTGGGGHAGYTGGAAG | GTYCCHACRTCATGVGTCTG | Zheng et al., 2019 |
| 40 | *nasA* | Nitrogen Cycle (Assimilatory N reduction) | CARCCNAAYGCNATGGG | ATNGTRTGCCAYTGRTC | Zheng et al., 2019 |
| 41 | *narG* | Nitrogen Cycle (Denitrification) | TAYGTSGGGCAGGARAAACTG | CGTAGAAGAAGCTGGTGCTGT | Zheng et al., 2019 |
| 42 | *nirK1* | Nitrogen Cycle (Denitrification) | GGMATGGTKCCSTGGCA | GCCTCGATCAGRTTRTGGTT | Zheng et al., 2019 |
| 43 | *nirK2* | Nitrogen Cycle (Denitrification) | ATGGCGCCATCATGGTNYTNCC | TCGAAGGCCTCGATNARRTTRTG | Zheng et al., 2019 |
| 44 | *nirK3* | Nitrogen Cycle (Denitrification) | TGCACATCGCCAACGGNATGTWYGG | GGCGCGGAAGATGSHRTGRTCNAC | Zheng et al., 2019 |
| 45 | *nirS1* | Nitrogen Cycle (Denitrification) | GTSAACGTSAAGGARACSGG | GASTTCGGRTGSGTCTTGA | Zheng et al., 2019 |
| 46 | *nirS2* | Nitrogen Cycle (Denitrification) | ATCGTCAACGTCAARGARACVGG | TTCGGGTGCGTCTTSABGAASAG | Zheng et al., 2019 |
| 47 | *nirS3* | Nitrogen Cycle (Denitrification) | TGGAGAACGCCGGNCARGTNTGG | GATGATGTCCACGGCNACRTANGG | Zheng et al., 2019 |
| 48 | *nosZ1* | Nitrogen Cycle (Denitrification) | CGCRACGGCAASAAGGTSMSSGT | CAKRTGCAKSGCRTGGCAGAA | Zheng et al., 2019 |
| 49 | *nosZ2* | Nitrogen Cycle (Denitrification) | CGYTGTTCMTCGACAGCCAG | CGSACCTTSTTGCCSTYGCG | Zheng et al., 2019 |
| 50 | *napA* | Nitrogen Cycle (Dissimilatory N reduction) | CTGGACIATGGGYTTIAACCA | CCTTCYTTYTCIACCCACAT | Zheng et al., 2019 |
| 51 | *nifH* | Nitrogen Cycle (Fixation) | AAAGGYGGWATCGGYAARTCCACCAC | TGSGCYTTGTCYTCRCGGATBGGCAT | Zheng et al., 2019 |
| 52 | *gdhA* | Nitrogen Cycle (N Mineralization) | GCCATCGGYCCWTACAAGGG | ATGTCRCCNGCCGGAACGTC | Zheng et al., 2019 |
| 53 | *amoA1* | Nitrogen Cycle (Nitrification) | STAATGGTCTGGCTTAGACG | GCGGCCATCCATCTGTATGT | Zheng et al., 2019 |
| 54 | *amoA2* | Nitrogen Cycle (Nitrification) | GGGGTTTCTACTGGTGGT | CCCCTCKGSAAAGCCTTCTT | Zheng et al., 2019 |
| 55 | *amoB* | Nitrogen Cycle (Nitrification) | TGGTAYGACATKAWATGG | RCGSGGCARGAACATSGG | Zheng et al., 2019 |
| 56 | *hao* | Nitrogen Cycle (Nitrification) | TGTCACATGGGTGTAGACCA | ACCTGGAACATACCCAT | Zheng et al., 2019 |
| 57 | *nxrA* | Nitrogen Cycle (Nitrification) | CAGACCGACGTGTGCGAAAG | TCCACAAGGAACGGAAGGTC | Zheng et al., 2019 |
| 58 | *bpp* | Phophorus Cycle (Organic P Mineralization) | GACGCAGCCGAYGAYCCNGCNITNTGG | CAGGSCGCANRTCIACRTTRTT | Zheng et al., 2019 |
| 59 | *ppk* | Phosphorus Cycle (Inorganic P biosynthesis) | GACCCGAABGTRCTBGCSAT | TTATAATTNCCSGTNCCNA | Zheng et al., 2019 |
| 60 | *ppx* | Phosphorus Cycle (Inorganic P hydrolysis) | TGCATCTGGCGGACGGCCT | AGATCCGCCGCCAATATCA | Zheng et al., 2019 |
| 61 | *gcd* | Phosphorus Cycle (Inorganic P Solubilization) | ATCGCGTTCGGGCCGGACG | ATSAGRTTSAGCTCGTCCCA | Zheng et al., 2019 |
| 62 | *pqqC* | Phosphorus Cycle (Inorganic P solubilization) | AACCGCTTCTACTACCAG | GCGAACAGCTCGGTCAG | Zheng et al., 2019 |
| 63 | *cphy* | Phosphorus Cycle (Organic P Mineraization) | GTGGACCTRCGRMARGARWCICA | GTCCGACCATTGCCTGCYTCRCARTGRAMRTGIADCCA | Zheng et al., 2019 |
| 64 | *phnK* | Phosphorus Cycle (Organic P Mineraization) | CATCGTCGGCGAATCCGG | TGCTGCATGCCGCCGGAAAA | Zheng et al., 2019 |
| 65 | *phoD* | Phosphorus Cycle (Organic P Mineraization) | CAGTGGGACGACCACGAGGT | GAGGCCGATCGGCATGTCG | Zheng et al., 2019 |
| 66 | *phoX* | Phosphorus Cycle (Organic P Mineraization) | GARGAGAACWTCCACGGYTA | GATCTCGATGATRTGRCCRAAG | Zheng et al., 2019 |
| 67 | *16S* | Phylogenetic Marker | GTGCCAGCMGCCGCGG | CCGTCAATTCMTTTRAGTTT | Zheng et al., 2019 |
| 68 | *soxY* | Sulfur Cycle (Oxidation) | ATCGATGACAACCCCGTGCC | AGCTGGTCCATCTGCATGCCG | Zheng et al., 2019 |
| 69 | *yedZ* | Sulfur Cycle (Oxidation) | CTGCTGATCACGCTGGCCAT | GCGATGCAGCTTCTTCCAGCG | Zheng et al., 2019 |
| 70 | *apsA* | Sulfur Cycle (Reduction) | GGGYCTKTCCGCYATCAAYAC | ATCATGATCTGCCAGCGGCCGGA | Zheng et al., 2019 |
| 71 | *dsrA* | Sulfur Cycle (Reduction) | ACSCACTGGAAGCACG | GGTGGAGCCGTGCATGTT | Zheng et al., 2019 |
| 72 | *dsrB* | Sulfur Cycle (Reduction) | CAACATCGTYCAYACCCAGGG | GTGTAGCAGTTACCGCA | Zheng et al., 2019 |
| 73 | *16S* | Taxonomic | GGGTTGCGCTCGTTGC | ATGGYTGTCGTCAGCTCGTG | Yan *et al*., 2024 |
| *74* | *A cinetobacter baumannii(ompA)* | Taxonomic | TCTTGGTGGTCACTTGAAGC | ACTCTTGTGGTTGTGGAGCA | Yan *et al*., 2024 |
| 75 | *Bacteroidetes* | Taxonomic | GGARCATGTGGTTTAATTCGATGAT | AGCTGACGACAACCATGCAG | Yan *et al*., 2024 |
| 76 | *Enterococci(23S)* | Taxonomic | AGAAATTCCAAACGAACTTG | CAGTGCTCTACCTCCATCATT | Yan *et al*., 2024 |
| 77 | *Firmicutes* | Taxonomic | GGAGYATGTGGTTTAATTCGAAGCA | AGCTGACGACAACCATGCAC | Yan *et al*., 2024 |
| *78* | *Klebsiella pneumoniae (gltA)* | Taxonomic | ACGGCCGAATATGACGAATTC | AGAGTGATCTGCTCATGAA | Yan *et al*., 2024 |
| 79 | *mecA-Staphylococci* | Taxonomic | CGCAACGTTCAATTTAATTTTGTTAA | TGGTCTTTCTGCATTCCTGGA | Yan *et al*., 2024 |
| *80* | *Pseudomonas aeruginosa(ecfX)* | Taxonomic | AGCGTTCGTCCTGCACAAGT | TCCACCATGCTCAGGGAGAT | Yan *et al*., 2024 |
| 81 | *IS613* | Mobile Genetic Elements | AGGTTCGGACTCAATGCAACA | TTCAGCACATACCGCCTTGAT | Yan *et al*., 2024 |
| 82 | *tnpA* | Mobile Genetic Elements | GCCGCACTGTCGATTTTTATC | GCGGGATCTGCCACTTCTT | Yan *et al*., 2024 |
| 83 | *tnpA* | Mobile Genetic Elements | CCGATCACGGAAAGCTCAAG | GGCTCGCATGACTTCGAATC | Yan *et al*., 2024 |
| 84 | *tnpA* | Mobile Genetic Elements | GGGCGGGTCGATTGAAA | GTGGGCGGGATCTGCTT | Yan *et al*., 2024 |
| 85 | *tnpA* | Mobile Genetic Elements | CATCATCGGACGGACAGAATT | GTCGGAGATGTGGGTGTAGAAAGT | Yan *et al*., 2024 |
| 86 | *tnpA* | Mobile Genetic Elements | GAAACCGATGCTACAATATCCAATTT | CAGCACCGTTTGCAGTGTAAG | Yan *et al*., 2024 |
| 87 | *tnpA* | Mobile Genetic Elements | TGCAGATGGTTTAACCTTGGATATTT | TCGGTTCATCAAACTGCTTCAC | Yan *et al*., 2024 |
| 88 | *tnpA* | Mobile Genetic Elements | AATTGATGCGGACGGCTTAA | TCACCAAACTGTTTATGGAGTCGTT | Yan *et al*., 2024 |
| 89 | *Tp614* | Mobile Genetic Elements | GGAAATCAACGGCATCCAGTT | CATCCATGCGCTTTTGTCTCT | Yan *et al*., 2024 |
| 90 | *trfa* | Mobile Genetic Elements | ACGAAGAAATGGTTGTCCTGTTC | CGTCAGCTTGCGGTACTTCTC | Yan *et al*., 2024 |
| 91 | *pAKD1-IncP- 1β* | Mobile Genetic Elements | GGTAAGATTACCGATAAACT | GTTCGTGAAGAAGATGTA | Yan *et al*., 2024 |
| 92 | *PAMBL- 1-F_377old* | Mobile Genetic Elements | CAGGCTCTTAATGTGATA | TTATGCTCAATACTCGTG | Yan *et al*., 2024 |
| 93 | *pBS228-IncP- 1α* | Mobile Genetic Elements | CAATCCATCGACAATCAC | GACAATCAGCTACTTCAC | Yan *et al*., 2024 |
| 94 | *IncN_oriT* | Mobile Genetic Elements | TTGGGCTTCATAGTACCC | GTGTGATAGCGTGATTTATGC | Yan *et al*., 2024 |
| 95 | *IncN_rep* | Mobile Genetic Elements | AGTTCACCACCTACTCGCTCC | CAAGTTCTTCTGTTGGGATTCC | Yan *et al*., 2024 |
| 96 | *IncP_oriT* | Mobile Genetic Elements | CAGCCTCGCAGAGCAGGAT | CAGCCGGGCAGGATAGGTGAAGT | Yan *et al*., 2024 |
| 97 | *IncQ_oriT* | Mobile Genetic Elements | TTCGCGCTCGTTGTTCTTCGAGC | GCCGTTAGGCCAGTTTCTCG | Yan *et al*., 2024 |
| 98 | *IncW_trwAB* | Mobile Genetic Elements | AGCGTATGAAGCCCGTGAAGGG | AAAGATAAGCGGCAGGACAATAACG | Yan *et al*., 2024 |
| 99 | *tra-A* | Mobile Genetic Elements | AAGTGTTCAGGGTGCTTCTGCGC | GTCATGTACATGATGACCAAAA | Yan *et al*., 2024 |
| 100 | *traN* | Mobile Genetic Elements | GCTTGGCGGTCAGCAATT | TTAGGAATAACAATCGCTACACCTTTA | Yan *et al*., 2024 |
| 101 | *trb-C* | Mobile Genetic Elements | CGGYATWCCGSCSACRCTGCG | GCCACCTGYSBGCAGTCMCC | Yan *et al*., 2024 |
| 102 | *cro* | Mobile Genetic Elements | AGATGTTATCGACCACTTCGG | CCGCTTGGCGATAAGCG | Yan *et al*., 2024 |
| 103 | *EAE_05855* | Mobile Genetic Elements | CCCATCACCGCTGAACTGG | TGGGCGCTGCCATCTAAAC | Yan *et al*., 2024 |
| 104 | *IncHI2-smr0018* | Mobile Genetic Elements | ATAATGATTCACCGGGGTAG | CTTCAGGCTATCGTTTCG | Yan *et al*., 2024 |
| 105 | *IncI1_repI1* | Mobile Genetic Elements | CGAAAGCCGGACGGCAGAA | TCGTCGTTCCGCCAAGTTCGT | Yan *et al*., 2024 |
| 106 | *IncN_korA* | Mobile Genetic Elements | GGAACGTTTGTAYCTTGTATTG | ACTCACTATCTTCTGTTGATTG | Yan *et al*., 2024 |
| 107 | *IS1247* | Mobile Genetic Elements | CGGCCGTCACTGACCAA | TCGGCAGGTTGGTGACG | Yan *et al*., 2024 |
| 108 | *IS15DI* | Mobile Genetic Elements | CAATACCTTTGATGGTGGCGTAAG | CTTACGCCACCATCAAAGGTATTG | Yan *et al*., 2024 |
| 109 | *IS200* | Mobile Genetic Elements | CCAAATACCGAAGACAAGCGTTC | CCAAACTGCTCGTAAAGCATCAG | Yan *et al*., 2024 |
| 110 | *IS200* | Mobile Genetic Elements | GCACACCCGATGGAACTGTAA | TCGGCGGGATCTCCAGAAG | Yan *et al*., 2024 |
| 111 | *IS21-ISAs29* | Mobile Genetic Elements | GGTCCGTCAGGCACAAGTC | GGGATCGTATCGGCAAGCC | Yan *et al*., 2024 |
| 112 | *IS256* | Mobile Genetic Elements | CTTGCGCATCATTGGATGATG | AAGAACGGCTCCAATTAAGCG | Yan *et al*., 2024 |
| 113 | *IS26* | Mobile Genetic Elements | ATGGATGAAACCTACGTGAAGGTC | CGGTACTTAATCTGTCGGTGTTCA | Yan *et al*., 2024 |
| 114 | *IS3* | Mobile Genetic Elements | CGGTCTGAGCTTCGGGAA | AGAACTGTCACTCCGGTCTG | Yan *et al*., 2024 |
| 115 | *IS5/IS1182* | Mobile Genetic Elements | TTCTCGAAGAATCGCCATGGC | GCTTTGGATCGCTCCAATCGA | Yan *et al*., 2024 |
| 116 | *IS6/257* | Mobile Genetic Elements | ATATCGTGCCATTGATGCAGA | ACCATTGCTACCTTCGTTGAAG | Yan *et al*., 2024 |
| 117 | *IS6100* | Mobile Genetic Elements | CGCACCGGCTTGATCAGTA | CTGCCACGCTCAATACCGA | Yan *et al*., 2024 |
| 118 | *IS630* | Mobile Genetic Elements | CCGCCACCAGTGTGATGG | TTGGCGCTGACTGGATGC | Yan *et al*., 2024 |
| 119 | *IS91* | Mobile Genetic Elements | GGATGCCACTGCTGGTCA | ACAGTGGATACAGTATCTGCTGAG | Yan *et al*., 2024 |
| 120 | *ISCR1* | Mobile Genetic Elements | ATGGTTTCATGCGGGTT | CTGAGGGTGTGAGCGAG | Yan *et al*., 2024 |
| 121 | *ISEcp1* | Mobile Genetic Elements | CATGCTCTGCGGTCACTTC | GACGCACCTTCTTGATGACC | Yan *et al*., 2024 |
| 122 | *lncF_FIC* | Mobile Genetic Elements | GTGAACTGGCAGATGAGGAAGG | TTCTCCTCGTCGCCAAACTAGAT | Yan *et al*., 2024 |
| 123 | *mobA* | Mobile Genetic Elements | GCTTCCCGTAACGAGGTAGT | CCTTGAACGGTATCAGCACG | Yan *et al*., 2024 |
| 124 | *Tn3* | Mobile Genetic Elements | GCTGAGGTGTTCAGCTACATC | GCTGAGGTAGTCACAGGCATTC | Yan *et al*., 2024 |
| 125 | *TN5403* | Mobile Genetic Elements | AAGCGAATGGCGCGAAC | CGCGCAGGGTAAACTGC | Yan *et al*., 2024 |
| 126 | *intI1_337old* | Mobile Genetic Elements | GCCTTGATGTTACCCGAGAG | GATCGGTCGAATGCGTGT | Yan *et al*., 2024 |
| 127 | *intl2* | Mobile Genetic Elements | TGCTTTTCCCACCCTTACC | GACGGCTACCCTCTGTTATCTC | Yan *et al*., 2024 |
| 128 | *intl3_339old* | Mobile Genetic Elements | GCCACCACTTGTTTGAGGA | GGATGTCTGTGCCTGCTTG | Yan *et al*., 2024 |
| 129 | *IS1111* | Mobile Genetic Elements | GTCTTAAGGTGGGCTGCGTG | CCCCGAATCTCATTGATCAGC | Yan *et al*., 2024 |
| 130 | *IS1133* | Mobile Genetic Elements | GCAGCGTCGGGTTGGA | ACGCGTTCGAACAACTGTAATG | Yan *et al*., 2024 |
| 131 | *ISAba3-Acineto* | Mobile Genetic Elements | TCAGAGGCAGCGGTATACGA | GGTTGATTCAGTTAAAGTACGTAAAACTTT | Yan *et al*., 2024 |
| 132 | *ISEfm1-Entero* | Mobile Genetic Elements | AGGTGTCCATGACGTGAAAGT | TCCTTTGTCCCCTAGGATATTG | Yan *et al*., 2024 |
| 133 | *ISPps1-pseud* | Mobile Genetic Elements | CACACTGCAAAAACGCATCCT | TGTCTTTGGCGTCACAGTTCTC | Yan *et al*., 2024 |
| 134 | *ISSm2-Xanthob* | Mobile Genetic Elements | TGGATCGACCGGTTCCAT | GCTGACCGAGCTGTCCATGT | Yan *et al*., 2024 |
| 135 | *orf37-IS26* | Mobile Genetic Elements | GCCGGGTTGTGCAAATAGAC | TGGCAATCTGTCGCTGCTG | Yan *et al*., 2024 |
| 136 | *orf39-IS26* | Mobile Genetic Elements | GCGCGTCGAGCATCAATAG | CAGTTGTGCTGCTGGTGGTC | Yan *et al*., 2024 |
| 137 | *TN5* | Mobile Genetic Elements | CAGCATAAAAAATCCCGACAACA | CCCCGCAACAGACATACGT | Yan *et al*., 2024 |
| 138 | *arsA* | Heavy Metal Resistance | CAGGTCAGCCGCATCAACC | GCCTGAAACACGGCAATTTCTTC | Yan *et al*., 2024 |
| 139 | *cadC* | Heavy Metal Resistance | CGCTCTGTGTCAGGATGAAGAG | CTTTCTTATGTGCTAGGGCGATCA | Yan *et al*., 2024 |
| 140 | *copA* | Heavy Metal Resistance | TGCACCTGACVGGSCAYAT | GVACTTCRCGGAACATRCC | Yan *et al*., 2024 |
| 141 | *czcA* | Heavy Metal Resistance | GCCTTGTTCATCGGCGAAC | GGCAATGTCGCCTTCGTTC | Yan *et al*., 2024 |
| 142 | *merA-marko* | Heavy Metal Resistance | GTGCCGTCCAAGATCATG | GGTGGAAGTCCAGTAGGGTGA | Yan *et al*., 2024 |
| 143 | *pbrT* | Heavy Metal Resistance | GATGCGCACTGGGCTTG | TCGGAATATGCGGAAATGCG | Yan *et al*., 2024 |
| 144 | *pcoA* | Heavy Metal Resistance | TGGCGTATGGAGTTTCAATGC | GAATAATGCCGTGCCAGTGAA | Yan *et al*., 2024 |
| 145 | *silE* | Heavy Metal Resistance | GGTGGAAAGTCATCAGAGGATGA | CAAAGCCCAGCAAGGATGC | Yan *et al*., 2024 |
| 146 | *tcrB* | Heavy Metal Resistance | GTGCCGGAACTCAAGTAGCA | GCACCGACTGCTGGACTTAA | Yan *et al*., 2024 |
| 147 | *terW* | Heavy Metal Resistance | TCAAAGAGCTACGCGAGTCAT | CCTTCCCTGTGGACTCACC | Yan *et al*., 2024 |
| 148 | *mecA* | Antibiotic Resistance | GGTTACGGACAAGGTGAAATACTGAT | TGTCTTTTAATAAGTGAGGTGCGTTAATA | Yan *et al*., 2024 |
| 149 | *pbp* | Antibiotic Resistance | CCGGTGCCATTGGTTTAGA | AAAATAGCCGCCCCAAGATT | Yan *et al*., 2024 |
| 150 | *Pbp5* | Antibiotic Resistance | GGCGAACTTCTAATTAATCCTATCCA | CGCCGATGACATTCTTCTTATCTT | Yan *et al*., 2024 |
| 151 | *penA* | Antibiotic Resistance | AGACGGTAACGTATAACTTTTTGAAAGA | GCGTGTAGCCGGCAATG | Yan *et al*., 2024 |
| 152 | *ACC- 1* | Antibiotic Resistance | CACACAGCTGATGGCTTATCTAAAA | AATAAACGCGATGGGTTCCA | Yan *et al*., 2024 |
| 153 | *ACT beta-lactamase* | Antibiotic Resistance | AAGCCGCTCAAGCTGGA | GCCATATCCTGCACGTTGG | Yan *et al*., 2024 |
| 154 | *ADC beta-lactamase* | Antibiotic Resistance | GGTATGGCTGTGGGTGTTATTCA | AGGCAAGGTTACCACTTGTATACG | Yan *et al*., 2024 |
| 155 | *AmpC beta-lactamase* | Antibiotic Resistance | CAGGATCTGATGTGGGAGAACTA | TCGGGAACCATTTGTTGGC | Yan *et al*., 2024 |
| 156 | *BEL beta-lactamase* | Antibiotic Resistance | ATGTCCATGGCACAGACTGTG | CCTGTCTTGTCACCCGTTACC | Yan *et al*., 2024 |
| 157 | *bl1acc* | Antibiotic Resistance | TGTTATCCGTGATTACCTGTCTGG | CTCAGCGAGCCAACTTCAAATA | Yan *et al*., 2024 |
| 158 | *Bla1* | Antibiotic Resistance | GCAAGTTGAAGCGAAAGAAAAGA | TACCAGTATCAATCGCATATACACCTAA | Yan *et al*., 2024 |
| 159 | *BlaB beta-lactamase* | Antibiotic Resistance | CGTGCCGGAGGTCTTGAATA | GGGATAGTAAACCTGAAACTCGGA | Yan *et al*., 2024 |
| 160 | *blaSFO* | Antibiotic Resistance | CCGCCGCCATCCAGTA | GGGCCGCCAAGATGCT | Yan *et al*., 2024 |
| 161 | *blaZ beta-lactamase* | Antibiotic Resistance | GGAGATAAAGTAACAAATCCAGTTAGATATGA | TGCTTAATTTTCCATTTGCGATAAG | Yan *et al*., 2024 |
| 162 | *CARB beta-lactamase* | Antibiotic Resistance | TGATTTGAGGGATACGACAACTCC | CTGTAATACTCCGAGCACCAA | Yan *et al*., 2024 |
| 163 | *CARB-2* | Antibiotic Resistance | TTGTGACCTATTCCCCTGTAATAGAA | TGCGAAGCACGCATCATC | Yan *et al*., 2024 |
| 164 | *CcrA* | Antibiotic Resistance | GCAGCGTTGCTGGACACA | GTTCGGGATAAACGTGGTGACT | Yan *et al*., 2024 |
| 165 | *CcrA beta-lactamase* | Antibiotic Resistance | CACTGGCACGGCGATTGTA | CGGCAGCCAAACCACGATA | Yan *et al*., 2024 |
| 166 | *cepA beta-lactamase* | Antibiotic Resistance | AGTTGCGCAGAACAGTCCTCT | TCGTATCTTGCCCGTCGATAAT | Yan *et al*., 2024 |
| 167 | *CfxA beta-lactamase* | Antibiotic Resistance | TCATTCCTCGTTCAAGTTTTCAGA | TGCAGCACCAAGAGGAGATGT | Yan *et al*., 2024 |
| 168 | *class C beta-lactamase* | Antibiotic Resistance | CTGGCGCATACCTGGATTAC | GCCAGTTCAGCATCTCCCA | Yan *et al*., 2024 |
| 169 | *CMY beta-lactamase* | Antibiotic Resistance | AAAGCCTCATGGGTGCATAAA | ATAGCTTTTGTTTGCCAGCATC | Yan *et al*., 2024 |
| 170 | *CMY_MOX beta-lactamase* | Antibiotic Resistance | CTATGTCAATGTGCCGAAGCA | GGCTTGTCCTCTTTCGAATAGC | Yan *et al*., 2024 |
| 171 | *CphA beta-lactamase* | Antibiotic Resistance | GCGAGCTGCACAAGCTGAT | CGGCCCAGTCGCTCTTC | Yan *et al*., 2024 |
| 172 | *cphA2* | Antibiotic Resistance | GTAACGCCTACTGGAAGTCCA | CAGCTTCTCCTTGAGAATGCAG | Yan *et al*., 2024 |
| 173 | *CTX-M beta-* | Antibiotic Resistance | GCGATAACGTGGCGATGAAT | GTCGAGACGGAACGTTTCGT | Yan *et al*., 2024 |
| 174 | *CTX-M- 1_3_ 15* | Antibiotic Resistance | CGTACCGAGCCGACGTTAA | CAACCCAGGAAGCAGGCA | Yan *et al*., 2024 |
| 175 | *DHA beta-lactamase* | Antibiotic Resistance | TGGCCGCAGCAGAAAGA | CCGTTTTATGCACCCAGGAA | Yan *et al*., 2024 |
| 176 | *FOX beta-lactamase* | Antibiotic Resistance | CCTACGGCTATTCGAAGGAAGATAAG | CCGGATTGGCCTGGAAGC | Yan *et al*., 2024 |
| 177 | *GES beta-lactamase* | Antibiotic Resistance | GCAATGTGCTCAACGTTCAAG | GTGCCTGAGTCAATTCTTTCAAAG | Yan *et al*., 2024 |
| 178 | *GOB beta-lactamase* | Antibiotic Resistance | CTTGGGCTTGAATGCTCAGGT | TGTATGGTCGTAGTGAGCCTGA | Yan *et al*., 2024 |
| 179 | *HERA beta-lactamase* | Antibiotic Resistance | GGGCAACCGCATTCTGAC | GCATCTCCCACTTTATCGTCAC | Yan *et al*., 2024 |
| 180 | *IMI beta-lactamase* | Antibiotic Resistance | ACATCTACACCTGCAGCAGTA | AATCGCTTGGTACGCTAGCA | Yan *et al*., 2024 |
| 181 | *IMIR beta-lactamase* | Antibiotic Resistance | AGCCGGACTAGAGCTTCATG | GGCAGAACTCATCATCTGCAA | Yan *et al*., 2024 |
| 182 | *IMP beta-lactamase* | Antibiotic Resistance | GGAATAGAGTGGCTTAATTC | GGTTTAACAAAACAACCACC | Yan *et al*., 2024 |
| 183 | *IND beta-lactamase* | Antibiotic Resistance | CGCCTGTTAAACCCAACCTGT | CGCTCTGTCATCATGAGAGTGG | Yan *et al*., 2024 |
| 183 | *KPC beta-lactamase* | Antibiotic Resistance | GCCGCCAATTTGTTGCTGAA | GCCGGTCGTGTTTCCCTTT | Yan *et al*., 2024 |
| 185 | *L1 beta-lactamase* | Antibiotic Resistance | CACCGGGTTACCAGCTGAAG | GCGAAGCTGCGCTTGTAGTC | Yan *et al*., 2024 |
| 186 | *LEN beta-lactamase* | Antibiotic Resistance | TGTTCGCCTGTGTGTTATCTCC | GCAGCACTTTAAAGGTGCTCAC | Yan *et al*., 2024 |
| 187 | *MIR beta-lactamase* | Antibiotic Resistance | CGGTCTGCCGTTACAGGTG | AAAGACCCGCGTCGTCATG | Yan *et al*., 2024 |
| 188 | *NDM beta-lactamase* | Antibiotic Resistance | GGCCACACCAGTGACAATATC | CAGGCAGCCACCAAAAGC | Yan *et al*., 2024 |
| 189 | *OCH beta-lactamase* | Antibiotic Resistance | GGCGACTTGCGCCGTAT | TTTTCTGCTCGGCCATGAG | Yan *et al*., 2024 |
| 190 | *OXA- 10* | Antibiotic Resistance | CGACCGAGTATGTACCTGCTT | TCAAGTCCAATACGACGAGCT | Yan *et al*., 2024 |
| 191 | *OXY- 1- 1* | Antibiotic Resistance | AAAGGTGACCGCATTCGC | CCAGCGTCAGCTTGCG | Yan *et al*., 2024 |
| 192 | *OXY-2- 1* | Antibiotic Resistance | CGTTCAGGCGGCAGGTT | GCCGCGATATAAGATTTGAGAATT | Yan *et al*., 2024 |
| 193 | *PDC beta-lactamase* | Antibiotic Resistance | CGCCGTACAACCGGTGAT | GAAGTAATGCGGTTCTCCTTTCA | Yan *et al*., 2024 |
| 194 | *PER- 1* | Antibiotic Resistance | GCAAATGAAGCGCAGATGC | GACCACAGTACCAGCTGGTA | Yan *et al*., 2024 |
| 195 | *ROB- 1* | Antibiotic Resistance | GCAAAGGCATGACGATTGC | CGCGCTGTTGTCGCTAAA | Yan *et al*., 2024 |
| 196 | *SHV- 11* | Antibiotic Resistance | TTGACCGCTGGGAAACGG | TCCGGTCTTATCGGCGATAAAC | Yan *et al*., 2024 |
| 197 | *SME beta-lactamase* | Antibiotic Resistance | GAGGAAGACTTTGATGGGAGGATTG | CGCTATATTGCAATGCAGCAGAAG | Yan *et al*., 2024 |
| 198 | *TEM beta-lactamase* | Antibiotic Resistance | CGCCGCATACACTATTCTCAG | GCTTCATTCAGCTCCGGTTC | Yan *et al*., 2024 |
| 199 | *TLA beta-lactamase* | Antibiotic Resistance | ACACTTTGCCATTGCTGTTTATGT | TGCAAATTTCGGCAATAATCTTT | Yan *et al*., 2024 |
| 200 | *VEB beta-lactamase* | Antibiotic Resistance | CCCGATGCAAAGCGTTATG | GAAAGATTCCCTTTATCTATCTCAGACAA | Yan *et al*., 2024 |
| 201 | *VIM beta-lactamase* | Antibiotic Resistance | GCACTTCTCGCGGAGATTG | CGACGGTGATGCGTACGTT | Yan *et al*., 2024 |
| 202 | *tet(44)* | Antibiotic Resistance | CTCATGTAGATGCAGGAAAGACG | GTAACTGCTGCCTGAATTGTGA | Yan *et al*., 2024 |
| 203 | *tet32* | Antibiotic Resistance | CCATTACTTCGGACAACGGTAGA | CAATCTCTGTGAGGGCATTTAACA | Yan *et al*., 2024 |
| 204 | *tet36* | Antibiotic Resistance | AGAATACTCAGCAGAGGTCAGTTCCT | TGGTAGGTCGATAACCCGAAAAT | Yan *et al*., 2024 |
| 205 | *tetB(P)* | Antibiotic Resistance | TGGGCGACAGTAGGCTTAGAA | TGACCCTACTGAAACATTAGAAATATACCT | Yan *et al*., 2024 |
| 206 | *tetM* | Antibiotic Resistance | GGAGCGATTACAGAATTAGGAAGC | TCCATATGTCCTGGCGTGTC | Yan *et al*., 2024 |
| 207 | *tetO* | Antibiotic Resistance | CAACATTAACGGAAAGTTTATTGTATACCA | TTGACGCTCCAAATTCATTGTATC | Yan *et al*., 2024 |
| 208 | *tetQ* | Antibiotic Resistance | CGCCTCAGAAGTAAGTTCATACACTAAG | TCGTTCATGCGGATATTATCAGAAT | Yan *et al*., 2024 |
| 209 | *tetS* | Antibiotic Resistance | TTAAGGACAAACTTTCTGACGACATC | TGTCTCCCATTGTTCTGGTTCA | Yan *et al*., 2024 |
| 210 | *tetT* | Antibiotic Resistance | CCATATAGAGGTTCCACCAAATCC | TGACCCTATTGGTAGTGGTTCTATTG | Yan *et al*., 2024 |
| 211 | *tetW* | Antibiotic Resistance | ATGAACATTCCCACCGTTATCTTT | ATATCGGCGGAGAGCTTATCC | Yan *et al*., 2024 |
| 212 | *tetR* | Antibiotic Resistance | CCGTCAATGCGCTGATGAC | GCCAATCCATCGACAATCACC | Yan *et al*., 2024 |
| 213 | *tetX* | Antibiotic Resistance | AAATTTGTTACCGACACGGAAGTT | CATAGCTGAAAAAATCCAGGACAGTT | Yan *et al*., 2024 |
| 214 | *tet(38)* | Antibiotic Resistance | AAGCGACATTAGCCGGTTTAG | CTGCTCGTACTTAAGCCAAGG | Yan *et al*., 2024 |
| 215 | *tet(39)* | Antibiotic Resistance | TATAGCGGGTCCGGTAATAGGTG | CCATAACGATCCTGCCCATAGATAAC | Yan *et al*., 2024 |
| 216 | *tet(40)* | Antibiotic Resistance | CTGTCCGTGCGCAATATATCC | GGATATATTGCGCACGGACAG | Yan *et al*., 2024 |
| 217 | *tetA* | Antibiotic Resistance | CTCACCAGCCTGACCTCGAT | CACGTTGTTATAGAAGCCGCATAG | Yan *et al*., 2024 |
| 218 | *tetA(P)* | Antibiotic Resistance | GGAAACCTTAGTTCAGTGACTTGG | CCCATTTAACCACGCACTGAA | Yan *et al*., 2024 |
| 219 | *tetB* | Antibiotic Resistance | AGTGCGCTTTGGATGCTGTA | AGCCCCAGTAGCTCCTGTGA | Yan *et al*., 2024 |
| 220 | *tetC* | Antibiotic Resistance | ACTGGTAAGGTAAACGCCATTGTC | ATGCATAAACCAGCCATTGAGTAAG | Yan *et al*., 2024 |
| 221 | *tetD* | Antibiotic Resistance | AATTGCACTGCCTGCATTGC | GACAGATTGCCAGCAGCAGA | Yan *et al*., 2024 |
| 222 | *tetE* | Antibiotic Resistance | TTGGCGCTGTATGCAATGAT | CGACGACCTATGCGATCTGA | Yan *et al*., 2024 |
| 223 | *tetG* | Antibiotic Resistance | TCGCGTTCCTGCTTGCC | CCGCGAGCGACAAACCA | Yan *et al*., 2024 |
| 224 | *tetH* | Antibiotic Resistance | TTTGGGTCATCTTACCAGCATTAA | TTGCGCATTATCATCGACAGA | Yan *et al*., 2024 |
| 225 | *tetJ* | Antibiotic Resistance | CAGCGCCCATACGCCATTTA | CCTACTTCAGTAGTGTGCCAAGC | Yan *et al*., 2024 |
| 226 | *tetK* | Antibiotic Resistance | CAGCAGTCATTGGAAAATTATCTGATTATA | CCTTGTACTAACCTACCAAAAATCAAAATA | Yan *et al*., 2024 |
| 227 | *tetL* | Antibiotic Resistance | ATGGTTGTAGTTGCGCGCTATAT | ATCGCTGGACCGACTCCTT | Yan *et al*., 2024 |
| 228 | *tetPB* | Antibiotic Resistance | TGGCAAGACGAGTTTGACTGA | GATCGCTCCACTTCAGCGATAA | Yan *et al*., 2024 |
| 229 | *tetPB* | Antibiotic Resistance | TGGCAAGACGAGTTTGACTGA | GATCGCTCCACTTCAGCGATAA | Yan *et al*., 2024 |
| 230 | *sul1* | Antibiotic Resistance | GCCGATGAGATCAGACGTATT | CGCATAGCGCTGGGTTTC | Yan *et al*., 2024 |
| 231 | *sul2* | Antibiotic Resistance | TCATCTGCCAAACTCGTCGTTA | GTCAAAGAACGCCGCAATGT | Yan *et al*., 2024 |
| 232 | *sul3* | Antibiotic Resistance | CGCGCTCAAGGCAGATG | GGGAATGCCATCTGCCTTG | Yan *et al*., 2024 |
| 233 | *sulA_folP* | Antibiotic Resistance | CAGGCTCGTAAATTGATAGCAGAAG | CTTTCCTTGCGAATCGCTTT | Yan *et al*., 2024 |
| 234 | *arr-2* | Antibiotic Resistance | TTGGCGATTGGTGACTTGCTAA | ATCGTCTTCGAACGGTCCTG | Yan *et al*., 2024 |
| 235 | *arr-3* | Antibiotic Resistance | GATCGTCTTCGAACGGTCCTG | TTTGGCGATTGGTGACTTGCT | Yan *et al*., 2024 |
| 236 | *cat* | Antibiotic Resistance | ATCGGCCAGACTGGATATCGA | CACAGCTCCAGTTGCAACAAC | Yan *et al*., 2024 |
| 237 | *cat(pC221)* | Antibiotic Resistance | AATGACCGTATGCTGCAAGAA | TTTGCCTGCTATGGCATTCTG | Yan *et al*., 2024 |
| 238 | *catB2* | Antibiotic Resistance | GCTACTATTCCGGCTATTACCATG | GGGCTCCTCGTTCATGTAGA | Yan *et al*., 2024 |
| 239 | *catB3* | Antibiotic Resistance | GCACTCGATGCCTTCCAAAA | AGAGCCGATCCAAACGTCAT | Yan *et al*., 2024 |
| 240 | *catB8* | Antibiotic Resistance | CACTCGACGCCTTCCAAAG | CCGAGCCTATCCAGACATCATT | Yan *et al*., 2024 |
| 241 | *catB9* | Antibiotic Resistance | CACCTTATGAAGTGGTCGGTTCA | GTCTGATGAACACAGAGACTGCA | Yan *et al*., 2024 |
| 242 | *catI* | Antibiotic Resistance | GGGTGAGTTTCACCAGTTTTGATT | CACCTTGTCGCCTTGCGTATA | Yan *et al*., 2024 |
| 243 | *catII* | Antibiotic Resistance | CCTGGAACCGCAGAGAACA | CGGAACTCCGGAAACTGATTAAC | Yan *et al*., 2024 |
| 244 | *catIII* | Antibiotic Resistance | CTGATTGCTCAGGCCGTGAA | ATGAGTATGGGCAACTCAGTGC | Yan *et al*., 2024 |
| 245 | *catP* | Antibiotic Resistance | CCTTTGGACTGAGTGTAAGTCTGA | TAAAGCCATCGAAGGTTGACCA | Yan *et al*., 2024 |
| 246 | *catQ* | Antibiotic Resistance | AGGTGCACTTACAGTATGACTGC | AACGTGGGAAGTTCTCGTCATAC | Yan *et al*., 2024 |
| 247 | *cmlv* | Antibiotic Resistance | GCCCTCATCACCGTCTTCG | GGACGTTGGCGATGGAGAG | Yan *et al*., 2024 |
| 248 | *cmlA1* | Antibiotic Resistance | TAGGAAGCATCGGAACGTTGA | CAGACCGAGCACGACTGTTG | Yan *et al*., 2024 |
| 249 | *cmlA5* | Antibiotic Resistance | GCGCTCTTCGAGGATTCG | CCGCCCAAGCAGAAGTAGAC | Yan *et al*., 2024 |
| 250 | *cmx* | Antibiotic Resistance | GCGATCGCCATCCTCTGT | TCGACACGGAGCCTTGGT | Yan *et al*., 2024 |
| 251 | *fexA* | Antibiotic Resistance | TGGTGTGGCTGTTGCAATCTTA | CCAAGGTACAAAGCACCTTGG | Yan *et al*., 2024 |
| 252 | *floR* | Antibiotic Resistance | AACCCGCCCTCTGGATCA | GCCGTCGAGAAGAAGACGAA | Yan *et al*., 2024 |
| 253 | *bacA* | Antibiotic Resistance | ATCCGCGGCACCCTGA | CCTGCTTGATGGACTTGATGAAGA | Yan *et al*., 2024 |
| 254 | *MCR- 1. 1* | Antibiotic Resistance | CACATCGACGGCGTATTCTG | CAACGAGCATACCGACATCG | Yan *et al*., 2024 |
| 255 | *MCR-2. 1* | Antibiotic Resistance | CGGCGTACTTTAAGCGTTATGATG | GCATTTGGCATACCATGCAGATAG | Yan *et al*., 2024 |
| 256 | *fabK* | Antibiotic Resistance | CAGGAGCAGGAAATCCAAGC | CCAGCTTCCATTCCTTCTGC | Yan *et al*., 2024 |
| 257 | *folA* | Antibiotic Resistance | CGAGCAGTTCCTGCCAAAG | CCCAGTCATCCGGTTCATAATC | Yan *et al*., 2024 |
| 258 | *nisB* | Antibiotic Resistance | GGGAGAGTTGCCGATGTTGTA | AGCCACTCGTTAAAGGGCAAT | Yan *et al*., 2024 |
| 259 | *SAT-4* | Antibiotic Resistance | GAATGGGCAAAGCATAAAAACTTG | CCGATTTTGAAACCACAATTATGATA | Yan *et al*., 2024 |
| 260 | *sugE* | Antibiotic Resistance | CTTAGTTATTGCTGGTCTGCTGGA | GCATCGGGTTAGCGGACTC | Yan *et al*., 2024 |
| 261 | *nimE* | Antibiotic Resistance | TGCGCCAAGATAGGGCATA | GTCGTGAATTCGGCAGGTTTA | Yan *et al*., 2024 |
| 262 | *lsaC* | Antibiotic Resistance | AAACGGCGTGAAAGTATCAGG | TTGTGGTGATGTAACGGATGC | Yan *et al*., 2024 |
| 263 | *mel* | Antibiotic Resistance | TAATTATCGCAGCAGCTGGTTC | GTTCCCAAACGGAGTATAAGAGTG | Yan *et al*., 2024 |
| 264 | *mel* | Antibiotic Resistance | GGCAAGCTAGGTGTTGAGC | ATTGCTCAACACCTAGCTTGC | Yan *et al*., 2024 |
| 265 | *msrA* | Antibiotic Resistance | CTGCTAACACAAGTACGATTCCAAAT | TCAAGTAAAGTTGTCTTACCTACACCATT | Yan *et al*., 2024 |
| 266 | *msrC* | Antibiotic Resistance | TCAGACCGGATCGGTTGTC | CCTATTTTTTGGAGTCTTCTCTCTAATGTT | Yan *et al*., 2024 |
| 267 | *msrE* | Antibiotic Resistance | CGGCAGATGGTCTGAGCTTAA | CGCACTCTTCCTGCATAAAGGA | Yan *et al*., 2024 |
| 268 | *optrA* | Antibiotic Resistance | GGTGGATGAAGTCCGTACGG | AGGTTAGACCTCCAAGAGCCA | Yan *et al*., 2024 |
| 269 | *vgaA* | Antibiotic Resistance | GGAAGCTATAGAGGCGTTTGAATC | CCGAAGGTTCAATACTCAATCGAC | Yan *et al*., 2024 |
| 270 | *vgaALC* | Antibiotic Resistance | GTGAAGATGTCTCGGGTACAATTG | GAAATACCAGGATTCCCATGCAC | Yan *et al*., 2024 |
| 271 | *vgaB* | Antibiotic Resistance | TAAAAGAGAATAAGGCGCAAGGA | TGTTTAGTAGCATGTTGCATTTTCC | Yan *et al*., 2024 |
| 272 | *acrR* | Antibiotic Resistance | GCGCTGGAGACACGACAAC | GCCTTGCTGCGAGAACAAA | Yan *et al*., 2024 |
| 273 | *marR* | Antibiotic Resistance | GCTGTTGATGACATTGCTCACA | CGGCGTACTGGTGAAGCTAAC | Yan *et al*., 2024 |
| 274 | *Cfr Group* | Antibiotic Resistance | GCAAAATTCAGAGCAAGTTACGAA | AAAATGACTCCCAACCTGCTTTAT | Yan *et al*., 2024 |
| 275 | *acrA* | Antibiotic Resistance | GGTCTATCACCCTACGCGCTATC | GCGCGCACGAACATACC | Yan *et al*., 2024 |
| 276 | *acrB* | Antibiotic Resistance | AGTCGGTGTTCGCCGTTAAC | CAAGGAAACGAACGCAATACC | Yan *et al*., 2024 |
| 277 | *acrF* | Antibiotic Resistance | GCGGCCAGGCACAAAA | TACGCTCTTCCCACGGTTTC | Yan *et al*., 2024 |
| 278 | *adeA* | Antibiotic Resistance | CAGTTCGAGCGCCTATTTCTG | CGCCCTGACCGACCAAT | Yan *et al*., 2024 |
| 279 | *adeI* | Antibiotic Resistance | CAGTCTGGTTTGCAGTAACCA | CACTCCTACAACAACAGGCAA | Yan *et al*., 2024 |
| 280 | *cefa_qacelta* | Antibiotic Resistance | TAGTTGGCGAAGTAATCGCAA | TGCGATGCCATAACCGATTATG | Yan *et al*., 2024 |
| 281 | *ceoA* | Antibiotic Resistance | ATCAACACGGACCAGGACAAG | GGAAAGTCCGCTCACGATGA | Yan *et al*., 2024 |
| 282 | *cmr* | Antibiotic Resistance | CGGCATCGTCAGTGGAATT | CGGTTCCGAAAAAGATGGAA | Yan *et al*., 2024 |
| 283 | *emrB_qacA* | Antibiotic Resistance | CTTTTCTCTAACCGTACATTATCTACGATAAA | AGAACGTAGCGACTGATAAAATGCT | Yan *et al*., 2024 |
| 284 | *emrD* | Antibiotic Resistance | CTCAGCAGTATGGTGGTAAGCATT | ACCAGGCGCCGAAGAAC | Yan *et al*., 2024 |
| 285 | *mdtA* | Antibiotic Resistance | ACAAGCCCAGGGCCAAC | CCTTAATGGTGCCTTCGGTTTC | Yan *et al*., 2024 |
| 286 | *mdtE* | Antibiotic Resistance | CGTCGGCGCACTCGTT | TCCAGACGTTGTACGGTAACCA | Yan *et al*., 2024 |
| 287 | *mdtG* | Antibiotic Resistance | TTCCAGCCGGTCAGCAA | GACATCTCCCGCGAGTTCG | Yan *et al*., 2024 |
| 288 | *MdtK* | Antibiotic Resistance | TCGGGCATCCCGTTTATGATC | GTAGGCTGCGCATAATACCCA | Yan *et al*., 2024 |
| 289 | *mepA* | Antibiotic Resistance | ATCGGTCGCTCTTCGTTCAC | ATAAATAGGATCGAGCTGCTGGAT | Yan *et al*., 2024 |
| 290 | *MexA* | Antibiotic Resistance | AGGACAACGCTATGCAACGAA | CCGGAAAGGGCCGAAAT | Yan *et al*., 2024 |
| 291 | *MexB* | Antibiotic Resistance | CTGGAGATCGACGACGAGAAG | GAAATCGTTGACGTAGCTGGA | Yan *et al*., 2024 |
| 292 | *MexE* | Antibiotic Resistance | GGTCAGCACCGACAAGGTCTAC | AGCTCGACGTACTTGAGGAACAC | Yan *et al*., 2024 |
| 293 | *mtrD* | Antibiotic Resistance | CGGAGTCCATCGACCATTTG | ATCGTCGGCAAGGAGAATCA | Yan *et al*., 2024 |
| 294 | *mtrE* | Antibiotic Resistance | CGATGTGTCGTTTTGGAAGGT | CCTGCACCATGATTCCTCAATA | Yan *et al*., 2024 |
| 295 | *multidrug resistance* | Antibiotic Resistance | AATTTTGCCGATTATTGCTGAAA | GATTGTCATCATTCGTTTATCACCAA | Yan *et al*., 2024 |
| 296 | *norA* | Antibiotic Resistance | ATCGCCGTTTGGTGGTACG | TCCACCAATCCCTGGTCCTAAA | Yan *et al*., 2024 |
| 297 | *oqxA* | Antibiotic Resistance | GAGTCAACCTACCTCCACTATCA | GCTGCGAGTTATCCAGCAG | Yan *et al*., 2024 |
| 298 | *qacF_H* | Antibiotic Resistance | TCGCAACATCCGCATTAAAA | ATGGATTTCAGAACCAGAGAAAGAAA | Yan *et al*., 2024 |
| 299 | *qacH_351* | Antibiotic Resistance | GTCGGTGTTGCTTATGCAGTCT | CAACCAGGCAATGGCTGTAA | Yan *et al*., 2024 |
| 300 | *tolC* | Antibiotic Resistance | GGCCGAGAACCTGATGCA | AGACTTACGCAATTCCGGGTTA | Yan *et al*., 2024 |
| 301 | *ttgA* | Antibiotic Resistance | ACGCCAATGCCAAACGATT | GTCACGGCGCAGCTTGA | Yan *et al*., 2024 |
| 302 | *ttgB* | Antibiotic Resistance | TCGCCCTGGATGTACACCTT | ACCATTGCCGACATCAACAAC | Yan *et al*., 2024 |
| 303 | *oprD* | Antibiotic Resistance | ATGAAGTGGAGCGCCATTG | GGCCACGGCGAACTGA | Yan *et al*., 2024 |
| 304 | *pica* | Antibiotic Resistance | GCAATCGAGGCGGTGTTC | TTGCCGCAGCCAATTCA | Yan *et al*., 2024 |
| 305 | *pikR2* | Antibiotic Resistance | TCGTGGGCCAGGTGAAGA | TTCCCCTTGCCGGTGAA | Yan *et al*., 2024 |
| 306 | *lmrA* | Antibiotic Resistance | TTCAGATGCAATGGCGTTTG | ATAATCGGGAACATAATGAGCATAACTAC | Yan *et al*., 2024 |
| 307 | *Erm(34)* | Antibiotic Resistance | AAAGCGGTTTACAAGCGTTTCG | GGGTGCTCTAGGGTTGTTTAGTG | Yan *et al*., 2024 |
| 308 | *Erm(35)* | Antibiotic Resistance | CCTTCAGTCAGAACCGGCAA | GCTGATTTGACAGTTGGTGGTG | Yan *et al*., 2024 |
| 309 | *Erm(36)* | Antibiotic Resistance | GGCGGACCGACTTGCAT | TCTGCGTTGACGACGGTTAC | Yan *et al*., 2024 |
| 310 | *Erm(42)* | Antibiotic Resistance | TGTTGAGATTGGGCCTGGA | CTAAGGGTGGGTTCTCACTATCTA | Yan *et al*., 2024 |
| 311 | *Erm(K)* | Antibiotic Resistance | GTTTGATATTGGCATTGTCAGAGAAA | ACCATTGCCGAGTCCACTTT | Yan *et al*., 2024 |
| 312 | *erm(O)* | Antibiotic Resistance | TGATGACGGCTCAGTGG | GTGCACCAGCGCCTGA | Yan *et al*., 2024 |
| 313 | *ErmA* | Antibiotic Resistance | TCGTTGAGAAGGGATTTGCGA | TTGCATGCTTCAAAGCCTGTC | Yan *et al*., 2024 |
| 314 | *ermA_ermTR* | Antibiotic Resistance | ACATTTTACCAAGGAACTTGTGGAA | GTGGCATGACATAAACCTTCATCA | Yan *et al*., 2024 |
| 315 | *ErmB* | Antibiotic Resistance | GAACACTAGGGTTGTTCTTGC | CTGGAACATCTGTGGTATGGC | Yan *et al*., 2024 |
| 316 | *ErmD* | Antibiotic Resistance | TTTCCGGACAGCATTTGATGC | TCCACTGCCAATACCTTACCG | Yan *et al*., 2024 |
| 317 | *ErmE* | Antibiotic Resistance | GTCACGCAGCTGGAGTTCG | CGGTGAAGCACAGCTCGAC | Yan *et al*., 2024 |
| 318 | *ErmF* | Antibiotic Resistance | CAGCTTTGGTTGAACATTTACGAA | AAATTCCTAAAATCACAACCGACAA | Yan *et al*., 2024 |
| 319 | *ErmG* | Antibiotic Resistance | CCCTTGAATTAGTACAGAGGT | GCAAACTCGTATTCCACGA | Yan *et al*., 2024 |
| 320 | *ErmH* | Antibiotic Resistance | GGAGTGAGGCTGACCGTAGAAG | ATCGGCGAAACGCACAAA | Yan *et al*., 2024 |
| 321 | *ErmQ* | Antibiotic Resistance | TGAAAGCCATGCGTCTGAC | TTCAGCTGGCAGCTTAAGC | Yan *et al*., 2024 |
| 322 | *ErmS* | Antibiotic Resistance | GAGTACGCCCGCAAACG | GCGTTCGATCCGGAGGA | Yan *et al*., 2024 |
| 323 | *ErmT* | Antibiotic Resistance | GTTCACTAGCACTATTTTTAATGACAGAAGT | GAAGGGTGTCTTTTTAATACAATTAACGA | Yan *et al*., 2024 |
| 324 | *ErmX* | Antibiotic Resistance | GCTCAGTGGTCCCCATGGT | ATCCCCCCGTCAACGTTT | Yan *et al*., 2024 |
| 325 | *ErmY* | Antibiotic Resistance | TTGTCTTTGAAAGTGAAGCAACAGT | TAACGCTAGAGAACGATTTGTATTGAG | Yan *et al*., 2024 |
| 326 | *EreA* | Antibiotic Resistance | GATAATTCTGCTGGCGCACA | GCAGGCGTGGTCACAAC | Yan *et al*., 2024 |
| 327 | *EreB* | Antibiotic Resistance | TCGTATATGGCGGGCGTAGTA | GGTCCAAGATGGGTGAATGCA | Yan *et al*., 2024 |
| 328 | *lnuA* | Antibiotic Resistance | TGACGCTCAACACACTCAAAAA | TTCATGCTTAAGTTCCATACGTGAA | Yan *et al*., 2024 |
| 329 | *lnuB* | Antibiotic Resistance | GGATCGTTTACCAAAGGAGAAGG | AGCATAGCCTTCGTATCAGGAA | Yan *et al*., 2024 |
| 330 | *lnuC* | Antibiotic Resistance | GGGTGTAGATGCTCTTCTTGGA | CTTTACCCGAAAGAGTTTCTACCG | Yan *et al*., 2024 |
| 331 | *lnuF* | Antibiotic Resistance | ATACCGGTCATTTCCACTTGGC | GCATCAGGCTGATGAGGTTCAA | Yan *et al*., 2024 |
| 332 | *mphA* | Antibiotic Resistance | TCAGCGGGATGATCGACTG | GAGGGCGTAGAGGGCGTA | Yan *et al*., 2024 |
| 333 | *mphB* | Antibiotic Resistance | CGCAGCGCTTGATCTTGTAG | TTACTGCATCCATACGCTGCTT | Yan *et al*., 2024 |
| 334 | *vatA* | Antibiotic Resistance | ATGAACGGAGCGAATCATCGG | CCATACCGATCCAAACGTCATTTC | Yan *et al*., 2024 |
| 335 | *vatB* | Antibiotic Resistance | GCAATTGTTGCTGCGAATTCA | GTGCTGACCAATCCCACCA | Yan *et al*., 2024 |
| 336 | *vatE* | Antibiotic Resistance | GACCGTCCTACCAGGCGTAA | TTGGATTGCCACCGACAATT | Yan *et al*., 2024 |
| 337 | *mef(B)* | Antibiotic Resistance | CCGATAGGCTTACTTGTTGCAG | AGTCCACTTGCGGTTTCATTG | Yan *et al*., 2024 |
| 338 | *oleC* | Antibiotic Resistance | CCCGGAGTCGATGTTCGA | GCCGAAGACGTACACGAACAG | Yan *et al*., 2024 |
| 339 | *vanA* | Antibiotic Resistance | GGGCTGTGAGGTCGGTTG | TTCAGTACAATGCGGCCGTTA | Yan *et al*., 2024 |
| 340 | *VanB* | Antibiotic Resistance | TTGTCGGCGAAGTGGATCA | AGCCTTTTTCCGGCTCGTT | Yan *et al*., 2024 |
| 341 | *vanC* | Antibiotic Resistance | CCTGCCACAATCGATCGTT | CGGCTTCATTCGGCTTGATA | Yan *et al*., 2024 |
| 342 | *vanC2_vanC3* | Antibiotic Resistance | TGACTGTCGGTGCTTGTGA | GATAGAGCAGCTGAGCTTGTTC | Yan *et al*., 2024 |
| 343 | *vanD* | Antibiotic Resistance | CAGAGGAACATAATGTTTCGATAAAATCT | GCCGGATTTTGTGATTCCAA | Yan *et al*., 2024 |
| 344 | *vanG* | Antibiotic Resistance | TGTTTCGCAGAACCGTGTCAA | CCCTGCACTGTTCCATCTTCTC | Yan *et al*., 2024 |
| 345 | *vanHB* | Antibiotic Resistance | GAGGTTTCCGAGGCGACAA | CTCTCGGCGGCAGTCGTAT | Yan *et al*., 2024 |
| 346 | *vanHD* | Antibiotic Resistance | GTGGCCGATTATACCGTCATG | CGCAGGTCATTCAGGCAAT | Yan *et al*., 2024 |
| 347 | *vanRA* | Antibiotic Resistance | CCCTTACTCCCACCGAGTTTT | TTCGTCGCCCCATATCTCAT | Yan *et al*., 2024 |
| 348 | *vanRB* | Antibiotic Resistance | GCCCTGTCGGATGACGAA | TTACATAGTCGTCTGCCTCTGCAT | Yan *et al*., 2024 |
| 349 | *vanRC* | Antibiotic Resistance | TGCGGGAAAAACTGAACGA | CCCCCCATACGGTTTTGATTA | Yan *et al*., 2024 |
| 350 | *vanRC4* | Antibiotic Resistance | AGTGCTTTGGCTTATCTCGAAAA | TCCGGCAGCATCACATCTAA | Yan *et al*., 2024 |
| 351 | *vanRD* | Antibiotic Resistance | TTATAATGGCAAGGATGCACTAAAGT | CGTCTACATCCGGAAGCATGA | Yan *et al*., 2024 |
| 352 | *vanSA* | Antibiotic Resistance | CGCGTCATGCTTTCAAAATTC | TCCGCAGAAAGCTCAATTTGTT | Yan *et al*., 2024 |
| 353 | *vanSB* | Antibiotic Resistance | GAAGATAAAGAGGGAAGCGTACTC | CCGAATTGTCAGCCCTTGATAA | Yan *et al*., 2024 |
| 354 | *vanSC* | Antibiotic Resistance | ATCAACTGCGGGAGAAAAGTC | TCCGCTGTTCCGCTTCTT | Yan *et al*., 2024 |
| 355 | *vanTC* | Antibiotic Resistance | ACAGTTGCCGCTGGTGAAG | CGTGGCTGGTCGATCAAAA | Yan *et al*., 2024 |
| 356 | *vanTE* | Antibiotic Resistance | GTGGTGCCAAGGAAGTTGCT | CGTAGCCACCGCAAAAAAAT | Yan *et al*., 2024 |
| 357 | *vanTG* | Antibiotic Resistance | CGTGTAGCCGTTCCGTTCTT | CGGCATTACAGGTATATCTGGAAA | Yan *et al*., 2024 |
| 358 | *vanWB* | Antibiotic Resistance | CGGACAAAGATACCCCCTATAAAG | AAATAGTAAATTGCTCATCTGGCACAT | Yan *et al*., 2024 |
| 359 | *vanXA* | Antibiotic Resistance | TCGTTGGGACGCTAAATATGC | GGACGGTAACCGTCCCATA | Yan *et al*., 2024 |
| 360 | *vanXB* | Antibiotic Resistance | AGGCACAAAATCGAAGATGCTT | GGGTATGGCTCATCAATCAACTT | Yan *et al*., 2024 |
| 361 | *vanYB* | Antibiotic Resistance | GGCTAAAGCGGAAGCAGAAA | GATATCCACAGCAAGACCAAGCT | Yan *et al*., 2024 |
| 362 | *vanYD* | Antibiotic Resistance | AAGGCGATACCCTGACTGTCA | ATTGCCGGACGGAAGCA | Yan *et al*., 2024 |
| 363 | *FosB* | Antibiotic Resistance | CTTGCAGGCCTATGGATTGC | TCTGTTCTCAAGTGTGCCAGTA | Yan *et al*., 2024 |
| 364 | *FosX* | Antibiotic Resistance | AGCTGGTTTGTGGATTTGCA | CCACACCGAGAGCTTTAATCCG | Yan *et al*., 2024 |
| 365 | *QnrA* | Antibiotic Resistance | AGGATTTCTCACGCCAGGATT | CCGCTTTCAATGAAACTGCAA | Yan *et al*., 2024 |
| 366 | *QnrB4* | Antibiotic Resistance | TCACCACCCGCACCTG | GGATATCTAAATCGCCCAGTTCC | Yan *et al*., 2024 |
| 367 | *QnrB46_47_48* | Antibiotic Resistance | CGACGTTCAGTGGTTCAGATCTC | GCCAAGCCGCTCCATGAG | Yan *et al*., 2024 |
| 368 | *qnrB* | Antibiotic Resistance | GCGACGTTCAGTGGTTCAGA | GCTGCTCGCCAGTCGAA | Yan *et al*., 2024 |
| 369 | *QnrD* | Antibiotic Resistance | CGCTGGAATGGCACTGTGA | GCTCTCCATCCAACTTCACTCC | Yan *et al*., 2024 |
| 370 | *QnrS1_S3_S5* | Antibiotic Resistance | CCACTTTGATGTCGCAGATCTTC | CCCTCTCCATATTGGCATAGGAAA | Yan *et al*., 2024 |
| 371 | *QnrS2* | Antibiotic Resistance | TCCCGAGCAAACTTTGCCAA | GGTGAGTCCCTATCCAGCGA | Yan *et al*., 2024 |
| 372 | *QnrVC1_VC3_VC6* | Antibiotic Resistance | CTCACATCAGGACTTGCAAGA | ATGAAGCATCTCGAAGATCAG | Yan *et al*., 2024 |
| 373 | *QnrVC4_VC5_VC7* | Antibiotic Resistance | TTCCTTTAAACGGGCAAACCTC | CGATACCTGATTCATGAAGCTAGC | Yan *et al*., 2024 |
| 374 | *mdtH* | Antibiotic Resistance | ATGCTGGCTGTACAAGTGATG | CACTCCAGCGGGCGATA | Yan *et al*., 2024 |
| 375 | *pmrA* | Antibiotic Resistance | TTTGCAGGTTTTGTTCCTAATGC | GCAGAGCCTGATTTCTCCTTTG | Yan *et al*., 2024 |
| 376 | *qacA_B* | Antibiotic Resistance | AAGGGCCACTGCATTAGCTG | CCAGTCCAATCATGCCTGCA | Yan *et al*., 2024 |
| 377 | *QepA 1 2* | Antibiotic Resistance | GGGCATCGCGCTGTTC | GCGCATCGGTGAAGCC | Yan *et al*., 2024 |
| 378 | *dfrA1* | Antibiotic Resistance | GGAATGGCCCTGATATTCCA | AGTCTTGCGTCCAACCAACAG | Yan *et al*., 2024 |
| 379 | *dfrA10* | Antibiotic Resistance | CTTCAACTATCACAGAGCACGAAG | TCTACCGGTACATACACATCAGC | Yan *et al*., 2024 |
| 380 | *dfrA12* | Antibiotic Resistance | CCTCTACCGAACCGTCACACA | GCGACAGCGTTGAAACAACTA | Yan *et al*., 2024 |
| 381 | *dfrA14* | Antibiotic Resistance | CGGATCATGTCATTGTTTCAGG | ATGTTAGAGGCGAAGTCTTGG | Yan *et al*., 2024 |
| 382 | *dfrA15* | Antibiotic Resistance | AGGCCGAAAGACTTTCGAGTC | TCACCTTCTGGCTCAATGTCG | Yan *et al*., 2024 |
| 383 | *dfrA17* | Antibiotic Resistance | CGGGAACGGCCCTGATATTCC | CGTGTTGCGACCGCATACTTTC | Yan *et al*., 2024 |
| 384 | *dfrA18* | Antibiotic Resistance | GGAGCGAATCAAGGAGAAAGGAA | GCAATGCGTTGATCGGTATTCTC | Yan *et al*., 2024 |
| 385 | *dfrA21* | Antibiotic Resistance | TTGTTTCAACGCTGTCGCA | GGTTTCGGTTGAGACAAGCTC | Yan *et al*., 2024 |
| 386 | *dfrA22* | Antibiotic Resistance | CAGCCGAACACGGCAAAG | CGGAGTGCGTGTACGTGA | Yan *et al*., 2024 |
| 387 | *dfrA25* | Antibiotic Resistance | TCAAACTGGACAGCGGCTA | GTCGATTGTCGACACATGCA | Yan *et al*., 2024 |
| 388 | *dfrA27* | Antibiotic Resistance | GCCGCTCAGGATCGGTA | GTCGAGATATGTAGCGTGTCG | Yan *et al*., 2024 |
| 389 | *dfrA5* | Antibiotic Resistance | CCATGGAGTGCCAAAGGTG | CACCTTTGGCACTCCATGG | Yan *et al*., 2024 |
| 390 | *dfrA7* | Antibiotic Resistance | GTAATCGGTAGTGGTCCTGA | ATCAGGACCACTACCGATTAC | Yan *et al*., 2024 |
| 391 | *dfrA8* | Antibiotic Resistance | GGTCGCACCTGCATCGTTA | AGCGCCACCAATGACGTAG | Yan *et al*., 2024 |
| 392 | *dfrB4* | Antibiotic Resistance | CGGTTCGCATTCCCATCAAA | CGCAGTCATGGGATAAATCTGG | Yan *et al*., 2024 |
| 393 | *dfrBmulti* | Antibiotic Resistance | ACCAAGGCAGAAGTGAAGTCA | GGTGAGCCTCAGACTCGAC | Yan *et al*., 2024 |
| 394 | *dfrC* | Antibiotic Resistance | GTCGCTCACGATAAACAAAGAGTC | CCCTTCATGGTGAAATGAAGCTTG | Yan *et al*., 2024 |
| 395 | *dfrG* | Antibiotic Resistance | TCAATCGGAAGAGCCTTACCTGA | TGGGCAAATACCTCATTCCATTCC | Yan *et al*., 2024 |
| 396 | *dfrK* | Antibiotic Resistance | TGCTGCGATGGATAAGAACAG | CTTCCAGGTAATGCTCTTCCG | Yan *et al*., 2024 |
| 397 | *AAC(3)-Ia* | Antibiotic Resistance | ACGTTCTGCCAAAGTTTGAG | ACTGCCGGATCGTCAC | Yan *et al*., 2024 |
| 398 | *AAC(3)-Ib* | Antibiotic Resistance | CAGCGAGACGTTCATCGC | CACGCTTCAGGTGGCTAATC | Yan *et al*., 2024 |
| 399 | *AAC(3)-Id* | Antibiotic Resistance | AGATAGTTATGCCCGCAACAA | ACGCGCTGCGCCTATA | Yan *et al*., 2024 |
| 400 | *AAC(3)-Iic* | Antibiotic Resistance | ACGGCATTCTCGATTGCTTT | CCGAGCTTCACGTAAGCATTT | Yan *et al*., 2024 |
| 401 | *AAC(3)-IId_IIa_Iie* | Antibiotic Resistance | CGATGGTCGCGGTTGGTC | TCGGCGTAGTGCAATGCG | Yan *et al*., 2024 |
| 402 | *AAC(3)-IV* | Antibiotic Resistance | CCAACACGACGCTGCATC | GCTGTCGCCACAATGTCG | Yan *et al*., 2024 |
| 403 | *AAC(3)-Via* | Antibiotic Resistance | GTGTCCGTCGCCAAGGA | GGTGACGGCCTTGTCGA | Yan *et al*., 2024 |
| 404 | *AAC(3)-Xa* | Antibiotic Resistance | GCAAGCGGTTCGTGACGTA | TCAGGTGCTCCTCGATCCAG | Yan *et al*., 2024 |
| 405 | *AAC(6')-Ib* | Antibiotic Resistance | CGTCGCCGAGCAACTTG | CGGTACCTTGCCTCTCAAACC | Yan *et al*., 2024 |
| 406 | *AAC(6')-Ie-APH(2'')-la* | Antibiotic Resistance | CCAAGAGCAATAAGGGCATACCAA | GCCACACTATCATAACCACTACCG | Yan *et al*., 2024 |
| 407 | *AAC(6')-Ig* | Antibiotic Resistance | GCGATGTTAGAAGCCTCAATTCG | CACACTTCGGCCTGTCGAA | Yan *et al*., 2024 |
| 408 | *AAC(6')-IIa* | Antibiotic Resistance | CGACCCGACTCCGAACAA | GCACGAATCCTGCCTTCTCA | Yan *et al*., 2024 |
| 409 | *AAC(6')-IIc* | Antibiotic Resistance | CAGTCTTTGGCTAATCCATCACAG | AACGAACCCGGCCTTCTC | Yan *et al*., 2024 |
| 410 | *AAC(6')-Ij* | Antibiotic Resistance | ATGCCTGTATCTGAATCCCTGATG | GGCAATCGCTTGTTGAGTATCTG | Yan *et al*., 2024 |
| 411 | *AAC(6')-Im* | Antibiotic Resistance | CGTGAGCATTATACAGAGCAATGG | CCATTTCCGTTCGTAGATATTGGC | Yan *et al*., 2024 |
| 412 | *AAC(6')-Ip* | Antibiotic Resistance | GGGAATTATCGGAATAGCTCTTGG | TTGGGCTGTTCTTCCTAGCTAA | Yan *et al*., 2024 |
| 413 | *AAC(6')-Ir* | Antibiotic Resistance | GCTATAACGATCAGCAGCAAG | CGCGATGCATGGCATGAC | Yan *et al*., 2024 |
| 414 | *AAC(6')-Is* | Antibiotic Resistance | AAGCTTACTCTGGCCTGATCATG | TGCCTGAACGTCGATATTCAGG | Yan *et al*., 2024 |
| 415 | *AAC(6')-Iv* | Antibiotic Resistance | TTGGCTTATACCGACACCCA | CCCGTTGCGATACCTGAAC | Yan *et al*., 2024 |
| 416 | *AAC(6')-Iw* | Antibiotic Resistance | TGCGTCAGTTACTTACACGAA | CCTGATGCATTGCATGACTGA | Yan *et al*., 2024 |
| 417 | *AAC(6')-Iy* | Antibiotic Resistance | GCCTCAATCCGCCACGATTA | ACGCGCTCTGTTTCCTCAAA | Yan *et al*., 2024 |
| 418 | *AAC(6')-Iz* | Antibiotic Resistance | TGCGCCATGACTACGTGAAC | GACTGTCCGAAGCCAGTTCG | Yan *et al*., 2024 |
| 419 | *aacA_aphD* | Antibiotic Resistance | AGAGCCTTGGGAAGATGAAGTTT | TTGATCCATACCATAGACTATCTCATCA | Yan *et al*., 2024 |
| 420 | *aacA43* | Antibiotic Resistance | CTTGGCCTACATTAGATTCAGCTC | GCTCTCAATCTTTGATAGGAGCAG | Yan *et al*., 2024 |
| 421 | *aadA* | Antibiotic Resistance | GTTGTGCACGACGACATCATT | GGCTCGAAGATACCTGCAAGA | Yan *et al*., 2024 |
| 422 | *aadA10* | Antibiotic Resistance | ACAGGCACTCAACGTCATCG | CGCGGAGAACTCTGCTTTGA | Yan *et al*., 2024 |
| 423 | *aadA16* | Antibiotic Resistance | ACGGTGGCCTGAAGCC | GAATTGCAGTTCCCGTCTGG | Yan *et al*., 2024 |
| 424 | *aadA17* | Antibiotic Resistance | TGTACGGCTCCGCAGTG | CACGGAATGATGTCGTCGTG | Yan *et al*., 2024 |
| 425 | *aadA2* | Antibiotic Resistance | CAATGACATTCTTGCGGGTATC | GACCTACCAAGGCAACGCTAT | Yan *et al*., 2024 |
| 426 | *aadA21* | Antibiotic Resistance | ACGGCTCCGCAGTGGAT | GGCCACAGTAACCAACAAATC | Yan *et al*., 2024 |
| 427 | *aadA5* | Antibiotic Resistance | ATCACGATCTTGCGATTTTGCT | CTGCGGATGGGCCTAGAAG | Yan *et al*., 2024 |
| 428 | *aadA6* | Antibiotic Resistance | CCATCGAGCGTCATCTGGAA | CCCGTCTGGCCGGATAAC | Yan *et al*., 2024 |
| 429 | *aadA7* | Antibiotic Resistance | CACTCCGCGCCTTGGA | TGTGGCGGGCTCGAAG | Yan *et al*., 2024 |
| 430 | *aadA7* | Antibiotic Resistance | CACTCCGCGCCTTGGA | TGTGGCGGGCTCGAAG | Yan *et al*., 2024 |
| 431 | *ANT(2'')-Ia* | Antibiotic Resistance | CCTGCTTGGTGGGCAGAC | CGGCACGCAAGACCTCAA | Yan *et al*., 2024 |
| 432 | *ANT(4')* | Antibiotic Resistance | CCGACAACATTTCTACCATCCTT | ACCGAAGCGCTCGTCGTATA | Yan *et al*., 2024 |
| 433 | *ANT(4')-Ia* | Antibiotic Resistance | GATGGCCGCTGACACATG | TCAACATTGCGCCATAGTGG | Yan *et al*., 2024 |
| 434 | *ANT(6)* | Antibiotic Resistance | TACCTTATTGCCCTTGGAAGAGTTA | GGAACTATGTCCCTTTTAATTCTACAATCT | Yan *et al*., 2024 |
| 435 | *ANT(6)-Ia* | Antibiotic Resistance | TCGCCATGAGCTGCTGA | CCTATCATACTCCGGATAGGCATA | Yan *et al*., 2024 |
| 436 | *ANT(6)-Ib* | Antibiotic Resistance | AGAACATCCGACAGCACGTTC | CCAACCTTCCATGAAATCATTCGC | Yan *et al*., 2024 |
| 437 | *APH(2'')-Iva* | Antibiotic Resistance | TGAGCAGTATCATAAGTTGAGTGAAAAG | GACAGAACAATCAATCTCTATGGAATG | Yan *et al*., 2024 |
| 438 | *APH(3')-Ia* | Antibiotic Resistance | TGAACAAGTCTGGAAAGAAATGCA | CCTATTAATTTCCCCTCGTCAAAAA | Yan *et al*., 2024 |
| 439 | *APH(3'')-Ia* | Antibiotic Resistance | TAACAGCGATCGCGTATTTCG | TCCGACTCGTCCAACATCAATA | Yan *et al*., 2024 |
| 440 | *APH(3')-Ib* | Antibiotic Resistance | AACAGGTTTGGGAGGCGATG | CGCAACAAGCCTCTCCTGAA | Yan *et al*., 2024 |
| 441 | *APH(3')-Via* | Antibiotic Resistance | TCTCATGGCGATATCACGGATAG | TTTCCTCCGATGCATCCTCTC | Yan *et al*., 2024 |
| 442 | *APH(3')-VIIa* | Antibiotic Resistance | CTCTCTCATGGAGATATGAGCGCTA | AATCCGGTTCAAGTCCCAACATG | Yan *et al*., 2024 |
| 443 | *APH(3')-VIIIa* | Antibiotic Resistance | TCGGTATCCCGGTTGTGAG | ACACGAGGTACGGGAATCC | Yan *et al*., 2024 |
| 444 | *APH(4)-Ia* | Antibiotic Resistance | CGCTCCCGATTCCGGAA | CACAGTTTGCCAGTGATACACA | Yan *et al*., 2024 |
| 445 | *APH(4)-Ib* | Antibiotic Resistance | GGGAACACCGTGCTCACC | GTTGGTCCCGTGCAGGTC | Yan *et al*., 2024 |
| 446 | *APH(6)-Ia* | Antibiotic Resistance | CGCTGGGAGCTGAAGAGG | AGCATCGTGCTGCTCTCC | Yan *et al*., 2024 |
| 447 | *APH(6)-Ic* | Antibiotic Resistance | CACGACAACGTGCTCGAC | CCGTCTTCGGCGAACCA | Yan *et al*., 2024 |
| 448 | *APH(6)-Id* | Antibiotic Resistance | GCTCGGTCGTGAGAACAATCT | CAATTTCGGTCGCCTGGTAGT | Yan *et al*., 2024 |
| 449 | *APH(9)-Ib* | Antibiotic Resistance | GCTATGTGCTGGTGGACTGG | GGAACCACTCGACGAACTCG | Yan *et al*., 2024 |
| 450 | *APH3-III* | Antibiotic Resistance | CAGAAGGCAATGTCATACCACTTG | GACAGCCGCTTAGCCGAA | Yan *et al*., 2024 |
| 451 | *APHA3* | Antibiotic Resistance | AAAAGCCCGAAGAGGAACTTG | CATCTTTCACAAAGATGTTGCTGTCT | Yan *et al*., 2024 |
| 452 | *apmA* | Antibiotic Resistance | GGCGCACATGCATTCATCA | CTATACTCCAGTCCCACCATTTGA | Yan *et al*., 2024 |
| 453 | *armA* | Antibiotic Resistance | TCTTCGACGAATGAAAGAGTCG | GCTAATGGATTGAAGCCACAACC | Yan *et al*., 2024 |
| 454 | *spec_aph* | Antibiotic Resistance | GGTGCTGATATGAATGCCTTTGG | CATTGGGCGCATCAATAAATGG | Yan *et al*., 2024 |
| 455 | *str* | Antibiotic Resistance | AATGAGTTTTGGAGTGTCTCAACGTA | AATCAAAACCCCTATTAAAGCCAAT | Yan *et al*., 2024 |
| 456 | *strA* | Antibiotic Resistance | CCGGTGGCATTTGAGAAAAA | GTGGCTCAACCTGCGAAAAG | Yan *et al*., 2024 |

**Supplementary Table S3.** Relationship among the relative gene copy number, absolute gene copy number, relative gene abundance and absolute gene abundance.

| Summary of Data Transformation:  $a=Relative 16S rRNA gene copy number--from HT-qPCR$  $b=Relative CNPS gene copy number--from HT-qPCR$  $c=Absolute copy number of the 16S rRNA \left( \frac{Copies}{\mu l} \right)--from conventional qPCR$  $d=DNA concentration \left( \frac{ng}{\mu l} \right)--from the Nanodrop Spectrophotometer$  $Relative gene abundance=\frac{b}{a} \left( 2 \right)$  $Absolute Abundance of CNPS genes \left( \frac{copies}{ng DNA} \right)=\frac{\left[ c\times(b/{a)} \right]}{d} \left( 3 \right)$  $Absolute abundance of CNPS genes \left( \frac{copies}{g soil} \right)=\frac{\frac{c\times\frac{b}{a}}{d}\times X}{0.25 g soil} \left( 4 \right)$  $Where X=d\times DNA Volume (\mu l);$ |
| --- |

**Supplementary Table S4.** The physicochemical properties of the soil samples

| Sample ID | MC (%) | LOI (%) | Soil pH | TC (mg/kg) | TN (mg/kg) | TP (mg/kg) |
| --- | --- | --- | --- | --- | --- | --- |
| S0001B | 59.3 | 29.61 | 4.58 | 199 | 6.40 | 1125 |
| S0002B | 58.3 | 26.95 | 4.52 | 198 | 5.50 | 1054 |
| S0003B | 58.5 | 30.58 | 4.65 | 185 | 8.00 | 914 |
| S0004B | 67.7 | 52.69 | 4.48 | 302 | 15.10 | 976 |
| S0005B | 57.7 | 31.75 | 4.75 | 195 | 7.80 | 909 |
| S0006B | 65.3 | 14.65 | 4.39 | 73 | 5.10 | 548 |
| S0007B | 65.7 | 53.57 | 4.44 | 303 | 15.30 | 1008 |
| S0008B | 65.8 | 52.58 | 4.45 | 278 | 14.20 | 994 |
| S0009B | 57.2 | 29.87 | 4.5 | 208 | 6.80 | 1137 |
| S00010B | 58.5 | 32.61 | 4.74 | 205 | 7.50 | 830 |

**Supplementary Table S5.** The Mobile Genetic Elements (MGEs) from soil samples collected at the Steed Pond area of SRS. The MGEs were mostly insertion sequences (54%) and plasmid replication proteins (14%), based on the total gene copy numbers per gram of soil.

| S/N | MGE | Copy number/g soil | Class of MGE | References |
| --- | --- | --- | --- | --- |
| 1 | *EAA-05855* | 7.00E+10 | Bacteriophage | Yan et al. (2020) |
| 2 | *IncL_rep1* | 7.1E+11 | Plasmid replication protein | Gebrie (2023) |
| 3 | *IncL_rep* | 3.1E+11 | Plasmid replication protein | Gomez-Simmonds et al. (2022) |
| 4 | *IncP_oriT* | 3.7E+11 | Origin of transfer (oriT) of IncP plasmids | Gomez-Simmonds et al. (2022) |
| 5 | *mob-A* | 2.5E+11 | Mobilization protein | Gomez-Simmonds et al. (2022) |
| 6 | *trb-C* | 8E+11 | Conjugative transfer protein | Horna et al. (2024) |
| 7 | *IS1111* | 4.8E+11 | Insertion sequences | Siddique et al. (2024) |
| 8 | *IS1247* | 6.8E+11 | Insertion sequences | Siddique et al. (2024) |
| 9 | *IS200-2* | 6.00E+10 | Insertion sequences | Siddique et al. (2024) |
| 10 | *IS21-ISAs29* | 4E+11 | Insertion sequences | Siddique et al. (2024) |
| 11 | *IS3* | 4.00E+10 | Insertion sequences | Yin et al. (2023) |
| 12 | *IS6100* | 4.9E+11 | Insertion sequences | Yin et al. (2023) |
| 13 | *IS630* | 5.8E+11 | Insertion sequences | Yin et al. (2023) |
| 14 | *ISCR1* | 1.1E+11 | ISCR element (rolling-circle transposable element) | Yin et al. (2023) |
| 15 | *ISEcp1* | 4.8E+11 | Insertion sequences | Yin et al. (2023) |
| 16 | *ISSm2-Xanthob* | 3.7E+11 | Insertion sequences | Yin et al. (2023) |
| 17 | *orf39-IS26* | 2E+11 | Insertion sequences | Gebrie (2023) |
| 18 | *TN5403* | 3.2E+11 | Transposon | Gebrie (2023) |
| 19 | *tnpA-1* | 1.9E+11 | Transposase gene | Gebrie (2023) |
| 20 | *tnPA-3* | 3.1E+11 | Transposase gene | Gebrie (2023) |

**Supplementary Table S6.** Mantel permutation test of the ARG, MGE, MRG, and soil properties.

| Call: ARG and MGE | Call: MGE and MRG |
| --- | --- |
| Mantel statistic r: 0.913 | Mantel statistic r: 0.7416 |
| Significance: 0.001 | Significance: 0.001 |
| Upper quantiles of permutations (null model):  90% 95% 97.5% 99%  0.358 0.443 0.524 0.639 | Upper quantiles of permutations (null model):  90% 95% 97.5% 99%  0.247 0.347 0.429 0.508 |
|  |  |
|  |  |
| Number of permutations: 999 | Number of permutations: 999 |
|  |  |
| Call: ARG and MRG | Call: ARG and Soil Physicochemical Properties |
| Mantel statistic r: 0.6983 | Mantel statistic r: -0.03928 |
| Significance: 0.001 | Significance: 0.502 |
| Upper quantiles of permutations (null model):  90% 95% 97.5% 99%  0.354 0.435 0.489 0.558 | \| Upper quantiles of permutations (null model):  90% 95% 97.5% 99%  0.247 0.347 0.429 0.508 \| \| --- \| |
| Number of permutations: 999 | Number of permutations: 999 |

References

Gebrie, A. **(2023).** Transposable elements as essential elements in the control of gene expression. *Mobile DNA* **14**, 9. doi:10.1186/s13100-023-00297-3

Gomez-Simmonds, A., Annavajhala, M. K., Tang, N., Rozenberg, F. D., Ahmad, M., Park, H., Lopatkin, A. J., and Uhlemann, A. C. **(2022).** Population structure of *bla_KPC_*-harbouring IncN plasmids at a New York City medical centre and evidence for multi-species horizontal transmission. *Journal of Antimicrobial Chemotherapy* **77**, 1873–1882. doi:10.1093/jac/dkac114

Horna, P., Weybright, M. J., Ferrari, M., Jungherz, D., Peng, Y., Akbar, Z., Ilca, F. T., Otteson, G. E., Seheult, J. N., Ortmann, J., Shi, M., Maciocia, P. M., Herling, M., Pule, M. A., and Olteanu, H. **(2024).** Dual T-cell constant β chain (TRBC)1 and TRBC2 staining for the identification of T-cell neoplasms by flow cytometry. *Blood Cancer Journal* **14**, 34. doi:10.1038/s41408-024-01002-0

Siddiquee, R., Pong, C. H., Hall, R. M., and Ataide, S. F. **(2024).** A programmable seekRNA guides target selection by IS*1111* and IS*110* type insertion sequences. *Nature Communications* **15**, 5235. doi:10.1038/s41467-024-49474-9

Yan, D., Han, Y., Zhong, M., Wen, H., An, Z., and Capo, E. **(2024).** Historical trajectories of antibiotic resistance genes assessed through sedimentary DNA analysis of a subtropical eutrophic lake. *Environment International* **186**, 108654. doi:10.1016/j.envint.2024.108654

Yang, X., Sun, H., Fan, R., Fu, S., Zhang, J., Matussek, A., Xiong, Y., and Bai, X. **(2020).** Genetic diversity of the intimin gene (*eae*) in non-O157 Shiga toxin-producing *Escherichia coli* strains in China. *Scientific Reports* **10**, 3275. doi:10.1038/s41598-020-60225-w

Yin, X., Li, Y., Liu, Y., Zheng, J., Yu, X., Li, Y., Achterberg, E. P., and Wang, X. **(2023).** Dietary exposure to sulfamethazine alters fish intestinal homeostasis and promotes resistance gene transfer. *Aquatic Toxicology* **264**, 106733. doi:10.1016/j.aquatox.2023.106733

Zheng, B. X., and Ding, K. **(2019).** Data on quantitative microbial elemental cycling (QMEC) primer design and validation. *Data in Brief* **23**, 103820. doi:10.1016/j.dib.2019.103820
